# Effectiveness of educational technology on human immunodeficiency virus/AIDS prevention: randomized clinical trial*

**DOI:** 10.1590/1518-8345.7352.4515

**Published:** 2025-03-31

**Authors:** Priscila Cabral Melo Holanda, Wilson Jorge Correia de Abreu, Francisca Márcia Pereira Linhares, Ryanne Carolynne Marques Gomes Mendes, Fábia Alexandra Pottes Alves, Tatiane Gomes Guedes

**Affiliations:** 1 Universidade Federal de Pernambuco, Departamento de Enfermagem, Recife, PE, Brazil.; 2 Scholarship holder at the Coordenação de Aperfeiçoamento de Pessoal de Nível Superior (CAPES), Brazil.; 3 Escola Superior de Enfermagem do Porto, Enfermagem, Porto, Portugal.

**Keywords:** Educational Technology, Nursing, Aged, HIV, Disease Prevention, Acquired Immunodeficiency Syndrome

## Abstract

to evaluate the effectiveness of the board game “*Mural de Risco*” (Risk Wall) on the prevention of the Human Immunodeficiency Virus/AIDS in the knowledge of people aged 50 and over in a school context.

randomized controlled trial, with two arms, carried out in 18 schools with 100 people in Youth and Adult Education. A validated illustrated instrument was used to assess knowledge. The educational intervention took place in groups of 3 to 5 participants, with the “*Mural de Risco*” game, in which the participants judged the images that represented a lot, little or no risk of HIV infection. McNemar’s test was applied to the distribution of correct answers.

there was a significant increase in the mean scores in the intervention group between baseline and the 30th day (p=0.001), which did not occur in the control group (p=0.953).

the game was effective in increasing the knowledge of people aged 50 and over about the prevention of the Human Immunodeficiency Virus/AIDS. Brazilian Registry of Clinical Trials: RBR-5w9tx9.

## Highlights

**(1)** In Brazil and around the world, there are high HIV/aids rates among older adults. **(2)** The board game is a low-cost, easily accessible educational resource. **(3)** The board game contains images of older adults in everyday situations. **(4)** The “*Mural de Risco*” (Risk Wall) game was validated by experts and evaluated by the target audience. **(5)** The game can be used by nurses in health education activities.

## Introduction

The increase in the number of cases of Acquired Immune Deficiency Syndrome (AIDS) intensifies the effects of social inequalities and mainly affects people in vulnerable situations. Worldwide, millions of lives are still at risk and affected by the Human Immunodeficiency Virus (HIV). The HIV/aids Epidemiological Bulletin 2023 shows an increase in the number of new HIV cases in people aged 50 and over, when comparing the periods 2012 to 2017 and 2018 to 2023, from 21,861 to 29,821 cases, respectively ^(1)^. The Joint United Nations Program on HIV/AIDS - UNAIDS - has adopted a strategy to include this specific target group in its actions to combat HIV/aids, in order to reduce the costs of health care in the long term ^(2)^.

UNAIDS also highlights worrying data on the global response to HIV for 2025: new infections could reach 1.2 million. It is reiterated that during the last two years - the pandemic caused by the new coronavirus and other global crises - progress against the HIV pandemic has been weakened and resources have dwindled, and therefore millions of lives are at risk. Globally, new HIV infections fell by just 3.6% between 2020 and 2021. Eastern Europe, Central Asia, the Middle East, North Africa and Latin America are experiencing significant increases in annual infection rates. Worldwide, the rate of new infections fell by only 3.6% between 2020 and 2021 ^(2)^.

In all Brazilian regions, the incidence of HIV/aids among people aged 50 and over has increased. In 2022, Pernambuco registered the second highest number of HIV cases in the Northeast ^(1)^.

There are many factors that expose this group, such as immunosenescence, which begins to manifest itself in different ways, gradually and continuously, usually after the age of 50; inadequate condom use; lack of emotional support; low education levels; little discussion about sexuality in aging, which leads to a lack of knowledge about HIV prevention; among others. It is therefore important to encourage the use of Pre-Exposure Prophylaxis (PrEP) and Post-Exposure Prophylaxis (PEP) in this population ^(3)^. It is also necessary to take into account lifestyle, socio-economic conditions and the various factors that can interfere with HIV infections ^(4)^.

Considering not only chronological age, but above all the concept of biological age, is another determining factor in caring for this target group. From this perspective, studies involving HIV prevention and people aged 50 and over are becoming increasingly important and prominent, since they contribute to knowledge about adherence to safe sexual behavior and, consequently, to the mitigation of Sexually Transmitted Infections (STIs) ^(5)^.

The failure to include people aged 50 and over in strategic plans and public policies aimed at the sexual health of middle-aged and older adults makes them even more vulnerable and has a direct impact on their visibility in terms of the HIV exposure/prevention relationship. This vulnerability may be associated with unprotected sex, the use of drugs for erectile dysfunction and the invisibility of the sexuality of people aged 50 and over. In relation to HIV/aids prevention for older adults in Brazil, there is only one guideline, from 2017, which mentions older adults and relates the particularities of this population to the implementation of the care network ^(6)^.

The epidemiological outline and the social reality of this public in the face of HIV raise the need to change the social and biological conception related to HIV/aids infection, while at the same time warning of the importance of investing in prevention actions at an earlier age ^(7)^.

In this context, it is worth highlighting nursing professionals and their contribution to HIV/aids at local and global level, with clinical and behavioral measures, in the production of care aimed at confronting HIV, in carrying out actions focused on qualified listening ^(8)^ and in the development of educational technologies ^(9)^.

Educational technology, a tool developed based on scientific knowledge, can be used as a material in the educational scenario, like the board game. A study that used this tool in health actions identified contributions to behavioral changes, such as healthy eating, smoking cessation and safe sex, as well as improvements in cognitive impairment and depression ^(10)^.

In the educational sphere, the school is an indispensable institution for educating individuals, in order to help them acquire knowledge that favors healthy behaviors, such as HIV/aids prevention. It is therefore a space for nurses to reach adults and older adults through the Brazilian Ministry of Education’s Youth and Adult Education (*Educação de Jovens e Adultos*, EJA) program. This action is anchored in the Brazilian Ministry of Health’s Health at School Program (*Programa Saúde na Escola*, PSE), a strategy for integrating health and education for the development of citizenship ^(11)^.

It is therefore important to carry out studies that test the effectiveness of board games, with a focus on acquiring safe knowledge, aimed at people aged 50 and over, in line with the HIV epidemiological profile of this specific group, adding to the trend of studies with an emphasis on combating HIV in young, sexually active populations or those with risky sexual practices. In this sense, board games could innovate, in a simple and playful way, HIV prevention education for people aged 50 and over. They will also be able to provide interactive experiences that are closer to the reality of this audience and thus demystify taboos related to HIV prevention, which are still so present in society.

In order to reinforce the usefulness and scientific-social relevance of educational technologies, the aim is to evaluate the effectiveness of the board game “Mural de Risco” on the knowledge of people aged 50 and over in a school context about HIV/aids prevention.

## Method

### Study design

This is a randomized controlled non-pharmacological clinical trial with two arms - intervention and control. The Consolidated Standards of Reporting Trials (CONSORT) ^(12)^ guidelines for parallel groups were followed and the study was registered in the Brazilian Registry of Clinical Trials (ReBEC): RBR-5w9tx9.

### Locus

All 18 schools (state and municipal) in Recife (PE), Brazil, which hosted this study were open during the evening, with four hours of classes a day. The EJA in Recife has more than 25,000 students enrolled in the evening shift. They complete the entire elementary school course, divided into five modules, over a mean period of five years.

### Period

The survey took place during the months of October and November 2021.

### Population

The choice of people aged 50 and over, including older adults, in this study is due to the significant growth in the number of aids cases in this group, the negligence regarding the inclusion of these people in HIV/aids prevention policies and the immunosenescence of this age group ^(13)^. The population consisted of 100 people aged 50 or over, allocated 50 to the Intervention Group (IG) and 50 to the Control Group (CG). The ERC was randomized by cluster, allocating people to groups (schools) rather than individually ^(14)^.

### Selection criteria

The inclusion criteria were: being aged 50 or over, being regularly enrolled in state or municipal schools that offered the EJA program and having studied in this program for at least six months. People aged 50 or over who were not attending classes during the data collection period were excluded. The criterion for discontinuation was people aged 50 or over who dropped out of school after the start of the survey.

### Definition of the sample

To determine the sample size, we used the sample calculation equation for two experimental proportions ^(15)^, 95% confidence level, 90% test power and with the expected prevalence of knowledge and lack of knowledge about HIV/aids prevention, we obtained a sample size of 45 people aged 50 or over for each group. Considering a possible loss of 10% (five people aged 50 or over), the number needed in each group was 50 observations, totaling 100 participants in the sample.

**Figure 1 -:**
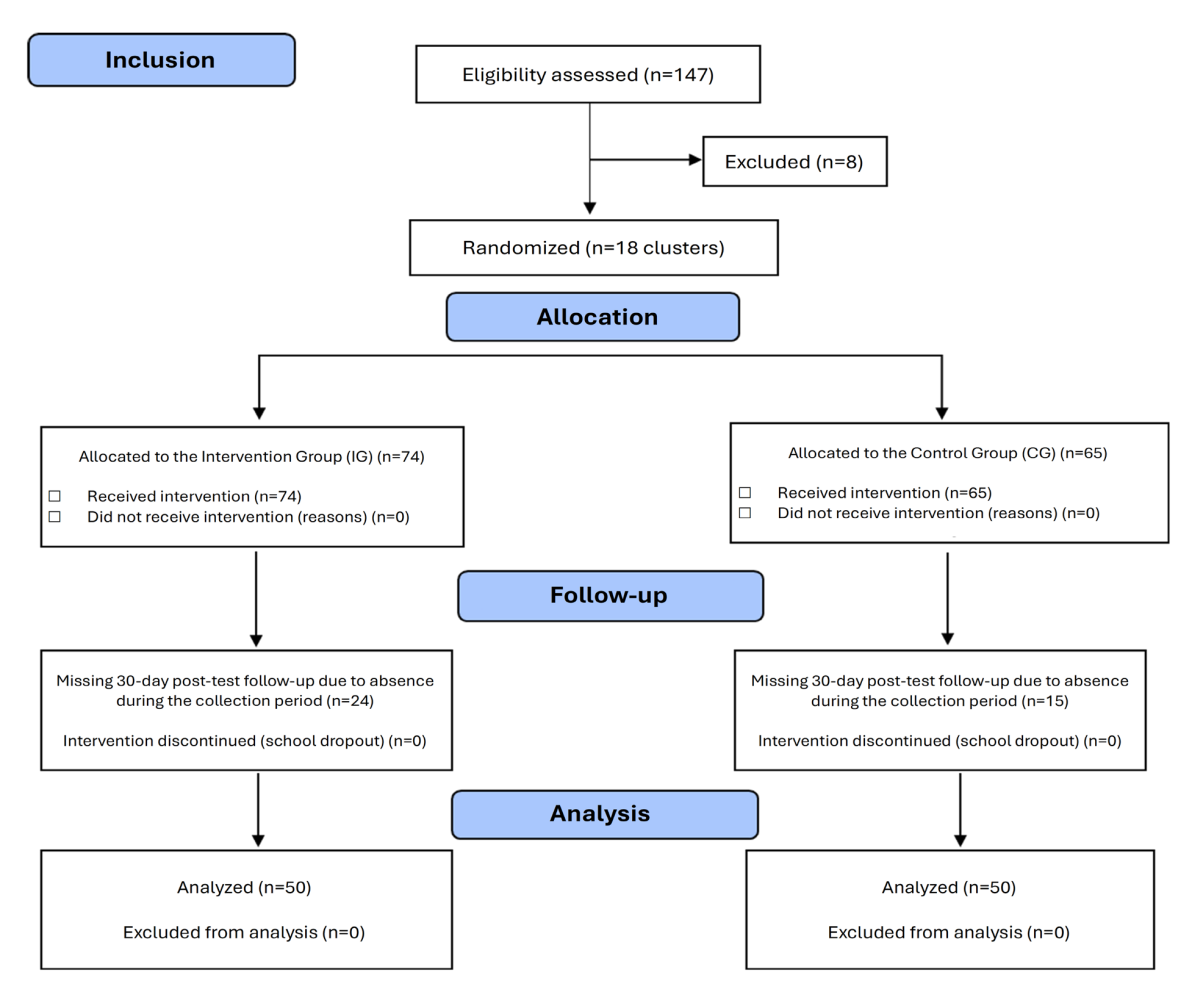
Data collection flowchart according to the Consolidated Standards of Reporting Trials ^(12)^. Recife, PE, Brazil, 2021

The sample was determined by convenience, according to the availability of students for data collection at the study sites. At first, a total of 215 students were identified as meeting the criteria. However, 68 were not attending classes at the time of data collection. Of these, 147 were eligible, of whom eight refused to take part. Thus, 139 were allocated, 74 to the IG and 65 to the CG. After the loss to follow-up (absence during the collection period), 50 students remained in the IG and 50 students in the CG (Figure 1).

Despite the similarity of the sample, EJA students aged 50 and over, the participants’ acquisition of knowledge during the data collection period in relation to HIV/aids prevention could be considered a potential confounding factor in the study.

### Study variables

Independent variables: sociodemographic variables were assessed: gender, age, marital status, religion, education (years of study) and lifestyle habits related to sexual health (how healthy they consider themselves, active sex life and physical activity).

Dependent variables: the dependent variable was knowledge of HIV/aids prevention, measured by the score obtained after the evaluation carried out during the initial interview and then on the thirtieth day for both the IG and CG.

### Data collection instrument

For data collection, an illustrated instrument was used, validated by health judges and evaluated by the target audience. The instrument has 12 short multiple-choice questions with options: “true”, “false” and “don’t know” ^(16)^. The questions involve everyday situations experienced by people aged 50 and over in a context of risk of HIV infection, such as: doing water aerobics, riding the bus, coughing, hugging, using sharp objects, among others ^(16)^.

The illustrations for the tool came from the “*Mural de Risco*” board, developed by game designers. The result of this illustrated instrument should be analyzed quantitatively, namely: nine or more correct questions indicates that there is adequate knowledge about the disease and different results reveal inadequate knowledge about HIV infection and aids. It should be noted that the answer “I don’t know” is classified as incorrect, i.e. it shows insufficient knowledge of the subject in question ^(16)^.

### Data collection

In order to evaluate the effectiveness of the board game entitled “*Mural de Risco*” (Risk Wall) on the knowledge of people aged 50 or over in a school setting about HIV/aids prevention, people were randomly and evenly allocated into groups IG and CG. In order to avoid contamination between the participants in the groups, which could occur if randomization was carried out individually because the students live in the same school, the type of randomization adopted in this study was by conglomerate or cluster ^(17)^.

Before contacting the students, the conglomerates were formed. This was done through simple random sampling to define the schools that made up conglomerates A (IG) and B (CG). To guarantee the confidentiality of the allocation, a sealed, opaque envelope was used with the names of the schools and the respective number of students. In order to compose each conglomerate, a draw was made: first for the schools in group A - IG, followed by the schools in group B - CG, so that each group allocated approximately the same number of students. According to the sample calculation, a number of students were counted in each group which, after allowing for losses, totaled at least 45 students in each group. This type of cluster or conglomerate randomization better adapts to research questions about health programs and their effects on the population ^(17)^.

The educational intervention with this board game, as well as the application of the pre- and post-tests, were carried out by a team of 12 previously trained and calibrated researchers through a pilot study. Initially, a baseline assessment was carried out, with the pre-test being administered to the IG and CG, followed by a second assessment with the post-test being administered immediately after the educational intervention in the IG, and also 30 days after the initial interview with the post-test being administered to both groups, in order to assess the knowledge retention of the study participants during this period.

It should be noted that the knowledge scores of the CG were measured on the basis of the natural exposure of this group (knowledge acquired habitually on a daily basis).

With the prior consent of the relevant school authorities, the students were invited to take part in the study during class time. They were informed about voluntary participation, emphasizing that the intervention would not affect the dynamics of the school and the content to be taught.

The steps below represent the dynamics of data collection:

Stage I: Introducing the researchers to the teaching team; requesting the attendance of the EJA students; carrying out the initial age screening; introducing the researchers in the classroom; explaining the research; and, once the students had expressed their interest in taking part, taking them to the space set aside by the school for data collection.

Stage II: Reaffirmation of acceptance to take part in the research by signing the Free and Informed Consent Form (FICF) in two copies; application of the illustrated instrument (pre-test) to assess baseline knowledge in both the IG and CG.

Stage III: Educational Intervention.

Shortly after completing the pre-test, the educational intervention was carried out with the game “*Mural de Risco*”. The game in question, developed by nurse researchers in the field of sexual health, has a user guide that is part of the illustrated instrument to assess the knowledge of older adults about HIV/aids prevention, both validated with a Content Validity Index (CVI = 0.90) and a Suitability Assessment of Materials score of 22 points for the game and 24 points for the user guide ^(18)^. The game was also evaluated by the target audience, with a Semantic Concordance Index of (SCI=0.80) ^(19)^. The explanatory guide provides important information on the dynamics of the game, namely: composition, purpose, target audience, step-by-step play and reflective points that can be addressed in the discussion of each image at the end of the game.

**Figure 2 -:**
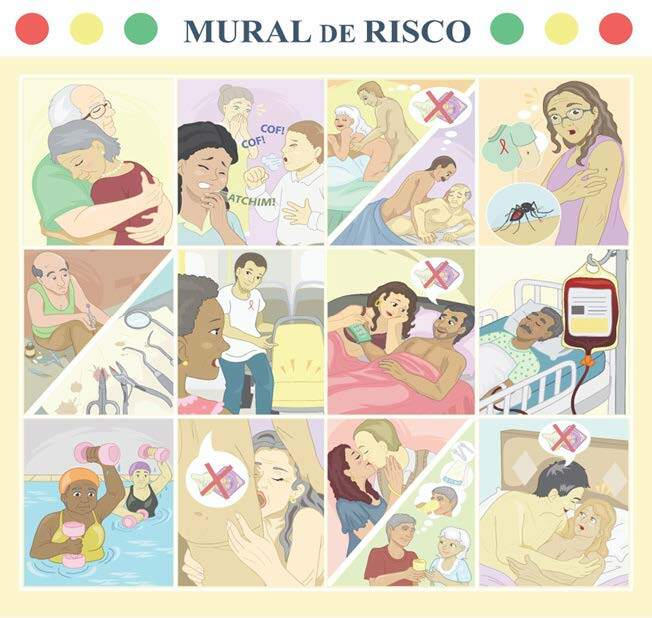
“*Mural de Risco*” game boards. Recife, PE, Brazil, 2021

The game board measures 1.00 x 1.00 meter, is hinged, contains 12 large, colorful vinyl images with Polyvinyl Chloride (PVC) application and transparent lamination for protection ^(18 , 20)^ (Figure 2).

The images in the game represent people aged 50 and over in different everyday situations that may or may not indicate a risk of HIV infection. The game comes with 36 magnetized pieces, 6 in red, 6 in yellow and 6 in green, which represent a high risk, low risk or no risk of HIV infection, respectively. The mediator organizes the board and the pieces, explains the objective of the game and promotes a moment of welcome and introduction; the group chooses one of the players to be the representative, everyone looks carefully at the board, analyzes each image and judges whether it represents a situation that indicates a lot, little or no risk of HIV infection.

The educational intervention took place in groups of 3 to 5 participants, lasting a mean of 30 minutes, mediated by collectors who had been previously trained to standardize the moment. After the initial moment of clarification about the dynamics of the game, the participants were arranged in a circle, with the board and the game pieces organized on a central table. Looking closely at the board, the participants analyzed the twelve images, one at a time, judging, by consensus, the risk of HIV infection, placing the red, yellow or green magnetized tile on the pictures on the wall. When all the images were overlaid with a magnetized piece, the mediator raised the board, turning it into a mural, allowing everyone to see the images. At the end of the game, the mediator discussed the successes and errors, answering any questions the group might have about HIV/aids prevention.

Stage IV: Immediate post-test applied to IG after the game.

Stage V: Reapplication of the post-test instrument after 30 days to analyze the knowledge of both the IG and CG.

### Data treatment and analysis

The data collected was compiled in Excel and analyzed using R software. The significance level adopted was 5% and the confidence interval was 95%. Descriptive statistics were used to summarize a set of observations. The Shapiro Wilk test was used to assess the normality of continuous data and define the choice of test (parametric or non-parametric). The chi-square and Fisher tests (categorical variables) and the Student’s t-test or Mann-Whitney test (continuous variables) were used to compare the groups. In order to compare the distribution of correct answers before and after the educational intervention, the McNemar non-parametric test was used to analyze the proportions between dichotomous variables in a paired fashion.

### Ethical aspects

The study was approved by the Research Ethics Committee under Opinion No. 4.258.634 and registered in the ReBEC database: RBR-5w9tx9. Participants signed the FICF in two copies, ensuring their anonymity, in accordance with the guidelines of Resolution 466/12 of the National Health Council ^(21)^.

## Results

**Figure 3 -:**
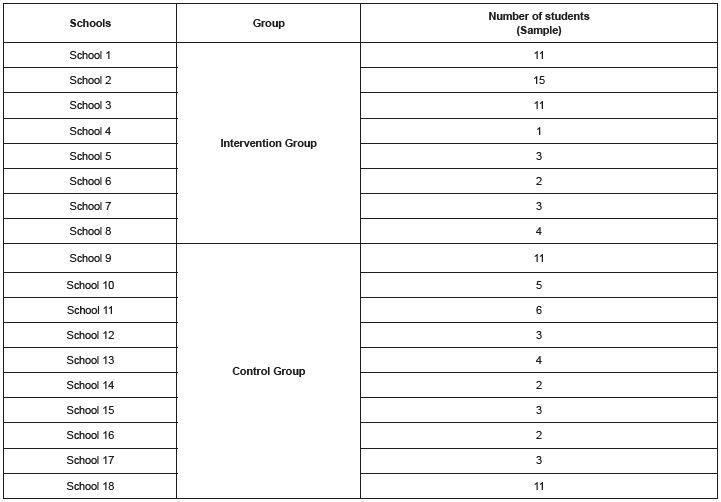
Number of students per school. Recife, PE, Brazil, 2021

Figure 3 shows the number of students, by school, who made up the IG (n=50) and CG (n=50) samples.

Table 1 shows the characterization of the participants.

**Table 1 -:** Sociodemographic characterization of the students (n* = 100). Recife, PE^†^, Brazil, 2021

**Characteristics**	General (n* = 100)	**CG** ^‡^ (n* = 50)	**IG** ^§^ (n* = 50)	**p-value** ^||^
**Gender**				
Female	83 (83.0%)	43 (86.0%)	40 (80.0%)	0.594a ^¶^ (v** = 0.08)
Male	17 (17.0%)	7 (14%,0)	10 (20.0%)	
**Age**				
Observations	100	50	50	0.025d ^††^ (r ^‡‡^ = 0.22)
Min-Max ^§§^	50-82	50-82	50-74	
Q1-Q3 ^||||^	53.75-64	54.25-66	53.25-61	
Median	59	60	56	
Mean	59.07	60.74	57.4	
Standard Deviation	6.86	7.42	5.85	
CV ^¶¶^	11.6%	12.2%	10.1%	
Normality (Shapiro-Wilk)	<0.001	0.052	0.008	
**Marital status**				
Married	34 (34.0%)	13 (26.0%)	21 (42.0%)	0.084a ^¶^ (v** = 0.29)
Divorced	12 (12.0%)	5 (10.0%)	7 (14.0%)	
Single	36 (36.0%)	18 (36.0%)	18 (36.0%)	
Stable Union	2 (2.0%)	2 (4.0%)	0 (0.0%)	
Widower/Widow	16 (16.0%)	12 (24.0%)	4 (8.0%)	
**Religion**				
Adventist	1 (1.0%)	1 (2.0%)	0 (0.0%)	0.895b***
Catholic	44 (44.0%)	20 (40.0%)	24 (48.0%)	
Spiritist	1 (1.0%)	1 (2.0%)	0 (0.0%)	
Evangelical	43 (43.0%)	22 (44%)	21 (42.0%)	
Afro-Brazilian	1 (1.0%)	1 (2.0%)	0 (0.0%)	
No religion	10 (10.0%)	5 (10.0%)	5 (10.0%)	
**Education in years of study**				
Observations	100	50	50	0.008d ^††^ (r ^‡‡^ = -0.27)
Min-Max ^§§^	1-10	1-8	1-10	
Q1-Q3 ^||||^	1-4	1-3	2-5	
Median	2.5	2	3	
Mean	3.03	2.5	3.56	
Standard Deviation	2.06	1.78	2.21	
CV ^¶¶^	68.0%	71.0%	61.9%	
Normality (Shapiro-Wilk)	<0.001	<0.001	<0.001	
**Active sex life**				
No	55 (55.0%)	31 (62.0%)	24 (48.0%)	0.228a ^¶^ (v** = 0.14)
Yes	45 (45.0%)	19 (38.0%)	26 (52.0%)	

*n = Sample; ^†^PE = Pernambuco; ^‡^CG = Control Group; ^§^IG = Intervention Group; ^||^p-value = Significance level; ^¶^a = Chi-squared test of independence; **v = Cramer’s v; ^††^d = Mann-Whitney test; ^‡‡^r = r-statistic; ^§§^Min-Max = Minimum and Maximum; ^||||^Q1-Q3 = First and third quartiles; ^¶¶^CV = Coefficient of Variation; ***b = Fisher’s Exact Test

Both groups were predominantly female. The mean education level in the control group was 2.5 (±1.78) years and in the intervention group 3.56 (±2.21) years.

The two groups were homogeneous, and the sociodemographic characteristics of the participants were similar, with a statistically significant difference only for age (p=0.025). There was no statistical significance (p=0.665) when comparing the baseline knowledge of CG (8.10) and IG (7.94) students about HIV/aids prevention, showing that participants in both groups had a similar level of knowledge before exposure.

Between the pre-intervention period and 30 days after the intervention, the IG showed substantially higher knowledge scores than the CG, with a statistically significant difference (p<0.001). While the mean knowledge score of students belonging to the CG was 8.06 (±2.24) points, that of the IG was 10.26 (±1.43) (Table 2).

**Table 2 -:** Mean knowledge scores of CG* and IG^†^ students on HIV/aids^‡^ prevention immediately after the educational intervention. Recife, PE^§^, Brazil, 2021

**Groups**					
**Period**	CG* (n=50)		IG ^†^ (n=50)		**p-value** ^||^
	**Mean ± SD** ^¶^	**CI** ^**^ **95%**	**Mean ± SD** ^¶^ **	**CI** ^**^ **95%**	
Moment 0 (Baseline)	8.10 ± ^¶^ 1.98	7.54 – 8.66	7.94 ± ^¶^ 2.04	7.36 – 8.52	0.665m ^††^
Immed. ^‡‡^ after	-	-	10.38 ± ^¶^ 1.28	10.02 – 10.74	-
After 30 days	8.06 ± ^¶^ 2.24	7.42 – 8.7	10.26 ± ^¶^ 1.43	9.85 – 10.67	<0.001m ^††^
Pre X Immed. ^‡‡^ after	-		<0.001w ^§§^		
Pre X Post 30 days	0.953w ^§§^		<0.001w ^§§^		

*CG = Control Group; ^†^IG = Intervention Group; ^‡^HIV/aids = Human Immunodeficiency Virus; ^§^PE = Pernambuco; ^||^p-value = Significance level; ^¶^SD = Standard Deviation; **CI = Confidence Interval; ^††^m = Man Whitney test; ^‡‡^Immed. = Immediately; ^§§^w = Wilcoxon

## Discussion

The increase and maintenance of knowledge after 30 days shown in this study is similar to the results of other studies that have also analyzed knowledge through the use of games in the teaching-learning process ^(22 - 23)^.

This study has had an impact on clinical nursing practice by offering a validated, low-cost technology that can be used in educational actions at any of the three levels of health care with the aim of preventing diseases or illnesses in people aged 50 and over.

One of the scenarios that educational interventions aimed at sex education can use is the school context. The choice of the 50 and over age group, EJA students, was based on the high rates of HIV infection in this population; the misconceptions of health and education professionals about sexuality for this group; the deficiency in the pedagogical training of EJA teachers to deal with sexual health issues; the fragility of the curriculum; and the debate and attention to sexual health, which is still very precarious in the school environment ^(24 - 25)^. In addition, increasing age implies a greater risk of diseases and health-related conditions, so individuals aged 50 or over show a more rapid progression towards immunosuppression, and there is also a greater appearance of opportunistic diseases, plus a greater likelihood of other comorbidities, which contributes to worsening health conditions ^(26)^.

Although there are barriers to working on the importance of HIV/aids prevention with the population in question, such as risky sexual behavior, precarious knowledge about sexuality and the difficulties or impossibilities of becoming pregnant, the educational intervention, using the “*Mural de Risco*” game, proved to be effective in increasing knowledge at baseline and after the intervention about HIV/aids prevention in people aged 50 and over in a school context.

However, while this result demonstrates positive evidence, it also prompts reflection on the importance of investing in health education actions that encourage safe sexual practices ^(27)^, since access to knowledge about HIV/aids prevention alone may not be enough or may simply not effectively lead to a change in unprotected sexual practices.

In relation to religiosity, almost half of the participants were Catholics. When assessing the spirituality, religiosity and beliefs of the public in question, a similar study found that spiritual and religious aspects are commonly present in the lives of these people. The fact that religion commonly associates sexuality only with the sexual act itself and not with understanding it in its entirety can be an inhibiting factor for sexual practice by this public, or even a risk factor because it can generate a feeling of repulsion ^(28)^.

During the course of the study, in addition to testing the effectiveness of the game, it also became clear that it is important to carry out health education activities on topics related to sexuality with the public in question, using educational technologies, since these are capable of stimulating cognition through the use of sight, hearing and touch, which has a direct impact on understanding the topic and motivating learning. It should also be emphasized that these actions should be encouraged and carried out by the teams in basic education.

As for years of schooling, similar data found in an investigation into the socio-epidemiological profile and autonomy of the long-lived ^(29)^, showed that 46.0% of the public had between one and four years of education. Another study, carried out in Northern Brazil ^(28)^ on HIV health literacy, found that the majority of people had no schooling or had only completed elementary school. It can be seen that the low literacy level favors increased vulnerability to HIV infection and, therefore, efforts must be made to give more attention and visibility to sexual health care for this public.

The sexual inactivity revealed by the target audience of this study is a reality that corroborates that identified in research which showed that factors such as the presence of comorbidities, bodily changes, sexual impotence and a distorted social view of sexuality mean that people aged 50 and over don’t feel comfortable or even feel guilty about expressing their sexual desires ^(30)^. However, during the games, the students verbalized their sexual desire and interest.

Given the physical, cultural and sexual exposure to HIV, it is important, given the sociodemographic profile data presented here, to clarify important aspects that can help improve the sexual health of older adults. These findings can contribute to nursing care by better planning and directing guidance, dialog, conduct and health literacy in this population. Therefore, an inclusive and targeted space must be offered, with qualified listening, judgment-free dialog, the establishment of bonds and evidence-based practices ^(31)^.

A systematic review of randomized clinical trials on games and gamified approaches in healthcare found that most of the primary studies resulted in a significant increase in knowledge through board games compared to other games. The meta-analysis carried out in this review showed a mean effect of board games on health-related knowledge (d* = 0.82, 95% confidence interval; CI [0.15-1.48]), with an increase in health knowledge in the majority of interventions that used board games (76.0%, n = 16) ^(32)^.

Ensuring the effectiveness of the “*Mural de Risco*” contributes to the body of knowledge of evidence-based nursing by including the patient in the care process, which is considered a challenge ^(33 - 34)^. The technology has therefore proved useful in disseminating knowledge about HIV prevention, while at the same time encouraging protagonism in self-care, and it is therefore believed that it has the potential to contribute to reducing HIV/AIDS rates among the public in question.

The study could therefore contribute to planned and targeted nursing care in terms of guidance, dialog and health behaviors for this population. With the use of the “*Mural de Risco*” (Risk Wall), a low-cost and easily accessible technology, by nurses during health education actions, it will be possible to offer an inclusive space aimed at the public aged 50 and over, with qualified listening, judgment-free dialog, the establishment of bonds and evidence-based practices.

From the above, and in view of the results that board games have on knowledge directed at health, such as knowledge about HIV and STIs, as well as their implications for the advancement of scientific knowledge in the area of health and nursing, it is suggested that the results discussed here are not exhausted by this study.

Limitations of the study include the potential bias of courtesy, with the application of the instruments carried out before and after a playful moment; the fact that the social, relational and emotional differences of the participants’ life stages were not taken into account when analyzing the data; and the assessment of knowledge retention after the proposed intervention carried out only immediately and in the medium term. It is therefore suggested that further analytical and/or qualitative studies be carried out to assess the phenomenon in question.

It should be noted that the study’s target audience was expanded to include people aged 50 and over, unlike the ReBEC protocol, which considered the inclusion of people aged 60 and over, due to the current epidemiological profile, which shows high rates of HIV in this specific population ^(1)^.

## Conclusion

The “Mural de Risco” board game was effective in increasing the knowledge of people aged 50 and over who took part in the educational interventions. It can therefore be used in nursing care for people aged 50 and over, whether in the school context or at health care levels, to enable this population to learn about ways of preventing the Acquired Immunodeficiency Virus/aids.

In order to identify research gaps that may not have been addressed, it is recommended that further research be carried out involving the subject in question and the use of this game in other contexts, such as people aged 50 or over with high education levels.

Finally, it should be noted that the game “Mural de Risco”, an educational technology registered with the national library (registration no.: 878.773 - book: 1712 - sheet: 481), can be replicated at low cost, including by hand, making learning about HIV/AIDS prevention more accessible and democratic.
