# Supplementary material for: Effectiveness of educational technology on human immunodeficiency virus/AIDS prevention: randomized clinical trial
Source: Rev Lat Am Enfermagem. 2025 Mar 31;33:e4515. doi: 10.1590/1518-8345.7352.4515 (PMC11960618; doi:10.1590/1518-8345.7352.4515)
Supplement: Translation

# Translation: Efetividade de tecnologia educacional sobre prevenção do vírus da imunodeficiência humana/aids: ensaio clínico randomizado*

**DOI:** 10.1590/1518-8345.7352.4516

**Authors:** Priscila Cabral Melo Holanda, Wilson Jorge Correia de Abreu, Francisca Márcia Pereira Linhares, Ryanne Carolynne Marques Gomes Mendes, Fábia Alexandra Pottes Alves, Tatiane Gomes Guedes

**Affiliations:** 1 Universidade Federal de Pernambuco, Departamento de Enfermagem, Recife, PE, Brasil.; 2 Bolsista da Coordenação de Aperfeiçoamento de Pessoal de Nível Superior (CAPES), Brasil.; 3 Escola Superior de Enfermagem do Porto, Enfermagem, Porto, Portugal.

**Keywords:** Tecnologia Educacional, Enfermagem, Pessoa Idosa, HIV, Prevenção de Doenças, Síndrome de Imunodeficiência Adquirida

## Abstract

avaliar a efetividade do jogo de tabuleiro “Mural de Risco” sobre prevenção do Vírus da Imunodeficiência Humana/aids no conhecimento de pessoas com 50 anos ou mais em contexto escolar.

ensaio randomizado controlado, com dois braços, realizado em 18 escolas com 100 pessoas da Educação de Jovens e Adultos. Para a avaliação do conhecimento, utilizou-se instrumento ilustrado validado. A intervenção educacional ocorreu em grupos de 3 a 5 participantes, com o jogo “Mural de Risco”, na qual os participantes julgaram as imagens que representavam muito, pouco ou nenhum risco para a infecção pelo HIV. Na distribuição dos acertos aplicou-se o teste de McNemar.

observou-se aumento significativo das médias dos escores no grupo intervenção entre o momento basal e o trigésimo dia (p=0,001), o que não ocorreu no grupo controle (p=0,953).

o jogo foi efetivo no aumento do conhecimento de pessoas com 50 anos ou mais sobre a prevenção do Vírus da Imunodeficiência Humana/aids. Registro Brasileiro de Ensaios Clínicos: RBR-5w9tx9.

## Destaques

**(1)** No Brasil e no mundo há altos índices de HIV/aids em pessoas idosas. **(2)** O jogo de tabuleiro é um recurso educacional de baixo custo e de fácil acesso. **(3)** O jogo de tabuleiro contém imagens de pessoas idosas em situações cotidianas. **(4)** O jogo “Mural de Risco” foi validado por especialistas e avaliado pelo público-alvo. **(5)** O jogo poderá ser utilizado por enfermeiros nas ações de educação em saúde.

## Introdução

O aumento do número de casos de Síndrome da Imunodeficiência Adquirida (SIDA/aids) intensifica os efeitos das desigualdades sociais e atinge principalmente as pessoas em situação de vulnerabilidade. Em nível mundial, milhões de vidas ainda estão em risco e são afetadas pelo Vírus da Imunodeficiência Humana (HIV). No Boletim Epidemiológico HIV/aids 2023, é possível identificar o aumento do número de novos registros de HIV em pessoas com 50 anos ou mais, quando comparados os períodos de 2012 a 2017 e 2018 a 2023, de 21.861 para 29.821 casos, respectivamente ^(1)^. Abranger esse público específico nas ações de combate ao HIV/aids, de forma a diminuir os custos dos cuidados de saúde a longo prazo, é uma estratégia do *Joint United Nations Programme on* HIV/aids - UNAIDS ^(2)^.

O UNAIDS destaca ainda dados preocupantes sobre a resposta global ao HIV para 2025: novas infecções podem chegar a 1,2 milhão. Reitera-se que durante os últimos dois anos - pandemia ocasionada pelo novo coronavírus e outras crises globais - houve enfraquecimento do progresso contra a pandemia do HIV e diminuição dos recursos e, portanto, milhões de vidas estão em risco. Em caráter mundial, as novas infecções pelo HIV diminuíram apenas 3,6% entre 2020 e 2021. A Europa Oriental, a Ásia Central, o Oriente Médio, o Norte da África e a América Latina vivem aumentos significativos nas taxas de infecções anuais. Mundialmente, o índice de novas infecções diminuiu somente 3,6% entre 2020 e 2021 ^(2)^.

Em todas as regiões do Brasil, tem aumentado a incidência de HIV/aids entre indivíduos com 50 anos ou mais. Em 2022, Pernambuco registrou o segundo maior número de casos de HIV do Nordeste ^(1)^.

Muitos são os fatores que expõem esse grupo, tais como a imunosenescência, que começa a se manifestar de diferentes maneiras, de forma gradual e contínua, geralmente após os 50 anos de idade; o uso de preservativo de forma inadequada; a falta de apoio emocional; a baixa escolaridade; pouca discussão sobre sexualidade no envelhecimento, que leva à falta de conhecimento acerca da prevenção do HIV; dentre outros. É importante, assim, o incentivo da utilização da Profilaxia Pré-Exposição (PrEP) e Profilaxia Pós-Exposição (PEP) nesta população ^(3)^. Ainda, é necessário valorizar o estilo de vida, as condições socioeconômicas e os diversos fatores que podem interferir nas infecções pelo HIV ^(4)^.

Considerar não apenas a idade cronológica, mas, sobretudo, a concepção de idade biológica é outro fator determinante no cuidado a esse público-alvo. Nesta perspectiva, estudos que envolvem a prevenção do HIV e as pessoas com 50 anos ou mais ganham cada vez mais importância e destaque, uma vez que contribuem para o conhecimento acerca da adesão a comportamentos sexuais seguros e, consequentemente, para a mitigação de Infecções Sexualmente Transmissíveis (IST) ^(5)^.

A não inclusão das pessoas com 50 anos ou mais nos planos estratégicos e nas políticas públicas destinadas à saúde sexual de adultos de meia idade e idosos as vulnerabiliza ainda mais e impacta diretamente na visibilidade destas quanto à relação exposição/prevenção do HIV. Essa vulnerabilidade pode estar associada ao sexo desprotegido, ao uso de drogas para disfunção erétil e à invisibilidade da sexualidade das pessoas com 50 anos ou mais. No que diz respeito ao público idoso, no Brasil, relacionada à prevenção do HIV/aids nessas pessoas, há apenas uma diretriz, de 2017, que menciona as pessoas idosas e faz relação com as particularidades dessa população para implementação da rede de cuidados ^(6)^.

O delineamento epidemiológico e a realidade social deste público frente ao HIV suscitam a necessidade de mudança quanto à concepção social e biológica relacionadas à infecção pelo HIV/aids, ao tempo que alertam para a importância do investimento em ações de prevenção cada vez mais precocemente ^(7)^.

Cabe destacar, nesse contexto, os profissionais de enfermagem e a contribuição destes, em nível local e global frente ao HIV/aids, com medidas clínico-assistenciais e comportamentais, na produção do cuidado direcionado ao enfrentamento ao HIV, na realização de ações com foco na escuta qualificada ^(8)^ e no desenvolvimento de tecnologias educacionais ^(9)^.

A tecnologia educacional, ferramenta desenvolvida baseada em conhecimentos científicos, pode ser utilizada como material no cenário educacional, a exemplo do jogo de tabuleiro. Estudo que utilizou essa ferramenta nas ações de saúde, identificou contribuições para modificações comportamentais, tais como a alimentação saudável, a cessação do tabagismo e o sexo seguro e, ainda, a melhora no comprometimento cognitivo e na depressão ^(10)^.

No âmbito educacional, a escola apresenta-se como instituição indispensável para a educação dos indivíduos, de forma a colaborar com a aquisição de conhecimentos que favoreçam comportamentos saudáveis, a exemplo da prevenção do HIV/aids. É, portanto, um espaço de atuação do enfermeiro para alcançar o público adulto e idoso por meio da Educação de Jovens e Adultos (EJA), do Ministério da Educação do Brasil. Tal atuação encontra-se ancorada no Programa Saúde na Escola (PSE), estratégia de integração da saúde e educação para o desenvolvimento da cidadania, do Ministério da Saúde do Brasil ^(11)^.

É importante, pois, a realização de estudos que testem a efetividade de jogos de tabuleiro, com foco na aquisição de conhecimentos seguros, direcionados a pessoas com 50 anos ou mais, em consonância com o perfil epidemiológico do HIV desse grupo específico, agregando-se a tendência de estudos com ênfase no combate ao HIV de populações jovens, sexualmente ativas ou com práticas sexuais de risco. Os jogos de tabuleiro, nesse sentido, poderão inovar, de forma simples e lúdica, à educação para a prevenção do HIV de pessoas com 50 anos ou mais. Poderão, ainda, oportunizar experiências interativas que se aproximam da realidade desse público e, assim, desmitificar tabus relacionados à prevenção do HIV, ainda tão presentes na sociedade.

Para reforçar a utilidade e relevância científico-social das tecnologias educacionais, objetiva-se avaliar a efetividade do jogo de tabuleiro “Mural de Risco” na prevenção do HIV/aids e no aumento do conhecimento sobre o tema entre pessoas com 50 anos ou mais, em um contexto escolar.

## Método

### Delineamento do estudo

Trata-se de um ensaio clínico randomizado (ERC) controlado não farmacológico, com dois braços - intervenção e controle. Seguiram-se as orientações do *Consolidated Standards of Reporting Trials* (CONSORT) ^(12)^ para grupos paralelos e o estudo foi registrado no Registro Brasileiro de Ensaios Clínicos (ReBEC): RBR-5w9tx9.

### Local

Todas as 18 escolas (estaduais e municipais) de Recife (PE), Brasil, que sediaram este estudo funcionavam no período noturno, com quatro horas diárias de aula. A EJA, da cidade do Recife, possui mais de 25 mil estudantes matriculados no turno da noite. Estes cumprem todo o ensino fundamental, dividido em cinco módulos, com período, em média, de cinco anos.

### Período

A pesquisa ocorreu durante os meses de outubro e novembro de 2021.

### População

A escolha de pessoas com 50 anos ou mais, incluindo os idosos, neste estudo deve-se ao fato do expressivo crescimento no número de casos de aids neste público, à negligência quanto à inserção destas pessoas nas políticas de prevenção do HIV/aids e à imunosenescência nesta faixa etária ^(13)^. A população foi constituída por 100 pessoas com 50 anos ou mais, alocadas 50 no Grupo Intervenção (GI) e 50 no Controle (GC). A randomização do ERC foi realizada por *cluster*, alocando as pessoas em grupos (escolas) e não individualmente ^(14)^.

### Critérios de seleção

Como critérios de inclusão considerou-se: ser pessoas com 50 anos ou mais, estar regularmente matriculado nas escolas estaduais ou municipais que possuíam a modalidade EJA e ter, no mínimo, seis meses de estudo nessa modalidade. Foram excluídas pessoas com 50 anos ou mais que não estavam frequentando as aulas no período de coleta de dados. Como critério de descontinuidade determinou-se pessoas com 50 anos ou mais que abandonaram o curso (evasão escolar) após o início da coleta.

### Definição da amostra

Para a determinação do tamanho da amostra utilizou-se a equação de cálculo de amostra para duas proporções experimentais ^(15)^, nível de confiança de 95%, poder do teste de 90% e com as prevalências esperadas de conhecimento e não conhecimento sobre prevenção do HIV/aids, obteve-se o tamanho amostral de 45 pessoas com 50 anos ou mais para cada grupo. Ao considerar possível perda de 10% (cinco pessoas com 50 anos ou mais), o número necessário em cada grupo foi de 50 observações, totalizando 100 participantes na amostra.

A amostra foi determinad a por conveniência, de acordo com a disponibilidade dos estudantes para a coleta de dados nos locais do estudo. No primeiro momento identificou-se que havia 215 estudantes que atendiam aos critérios determinados. No entanto, 68 não estavam frequentando as aulas no período de coleta de dados. Desse modo, 147 eram elegíveis, desses, oito pessoas recusaram a participação. Assim, 139 foram alocadas, sendo 74 no GI e 65 no GC. Após a perda de seguimento (ausência no período de coleta), restaram 50 estudantes no GI e 50 estudantes no GC (Figura 1).

Apesar da semelhança da amostra, estudantes da EJA com 50 anos ou mais, a aquisição de conhecimento dos participantes durante o período da coleta de dados em relação à prevenção do HIV/aids, pode ser considerado um fator potencial de confundimento do estudo.

**Figura 1 -:** Fluxograma de coleta de dados conforme *Consolidated Standards of Reporting Trials* ^(12)^. Recife, PE, Brasil, 2021

### Variáveis do estudo

Variáveis independentes: foram avaliadas as variáveis sociodemográficas: sexo, idade, estado civil, religião, escolaridade (anos de estudo) e hábitos de vida relacionados à saúde sexual (como considera a saúde, vida sexual ativa e prática de atividade física).

Variáveis dependentes: considerou-se como variável dependente o conhecimento sobre prevenção do HIV/aids, mensurado por meio do *score* obtido após a avaliação realizada durante a entrevista inicial e, posteriormente, no trigésimo dia, tanto para o GI como para o GC.

### Instrumento de coleta de dados

Para a coleta de dados utilizou-se instrumento ilustrado, validado por juízes da área da saúde e avaliado pelo público-alvo. O instrumento possui 12 questionamentos curtos de múltipla escolha com opções: “verdadeiro”, “falso” e “não sei” ^(16)^. Os questionamentos envolvem situações cotidianas vivenciadas pelo público com 50 anos ou mais em contexto de risco de infecção pelo HIV, a exemplo de: fazer hidroginástica, andar de ônibus, tossir, abraçar, fazer uso de objetos perfurocortantes, dentre outras ^(16)^.

As ilustrações do instrumento foram provenientes do tabuleiro “Mural de Risco”, desenvolvidas por *designers* de jogos. O resultado do referido instrumento ilustrado deve ser analisado quantitativamente, a saber: nove ou mais questões corretas indicam que há conhecimento adequado acerca da doença, e resultados diferentes revelam conhecimento inadequado sobre a infecção pelo HIV e aids. Destaca-se que a resposta “não sei” é classificada como incorreta, ou seja, demonstra a insuficiência de conhecimento sobre o assunto em questão ^(16)^.

### Coleta de dados

Para avaliar a efetividade do jogo de tabuleiro intitulado “Mural de Risco” no conhecimento de pessoas com 50 anos ou mais em contexto escolar sobre a prevenção do HIV/aids as pessoas foram alocadas, de forma aleatória e uniforme, compondo o GI e o GC. No intuito de evitar a contaminação entre os participantes dos grupos, fato que poderia ocorrer se a randomização fosse realizada individualmente por se tratar de estudantes que convivem no mesmo local de ensino, o tipo de randomização adotado neste estudo foi por conglomerado ou *cluster* ^(17)^.

Previamente ao contato com os estudantes, houve a formação dos conglomerados. Esta ocorreu por meio de amostragem aleatória simples para definição das escolas que integraram os conglomerados A (GI) e B (GC). Para garantir o sigilo de alocação, utilizou-se envelope selado e opaco com os nomes das escolas e o respectivo quantitativo de estudantes. A fim de compor cada conglomerado, realizou-se o sorteio: primeiro das escolas do grupo A - GI, seguido das escolas do grupo B - GC, de modo que cada grupo alocasse, aproximadamente, o mesmo quantitativo de estudantes. Conforme o cálculo amostral, contabilizou-se em cada grupo um quantitativo de estudantes que, após previsão das perdas, totalizasse pelo menos 45 estudantes em cada grupo. Esse tipo de randomização por *cluster* ou conglomerados adapta melhor as questões de pesquisas sobre programas de saúde e seus efeitos na população ^(17)^.

A intervenção educacional com o referido jogo de tabuleiro, bem como a aplicação dos pré e pós-testes, foram realizadas por uma equipe de 12 pesquisadores previamente treinados e calibrados, por meio do estudo-piloto. Inicialmente foi realizada uma avaliação basal, com aplicação do pré-teste no GI e GC, seguida de uma segunda avaliação com aplicação dos pós-teste logo após a intervenção educacional no GI e, ainda, 30 dias após a entrevista inicial com a aplicação do pós-teste em ambos os grupos, para avaliar a retenção de conhecimento dos participantes do estudo nesse período.

Destaca-se que os escores de conhecimento do GC foram medidos com base na exposição natural deste grupo (conhecimentos adquiridos habitualmente no dia a dia).

Com a anuência prévia das autoridades escolares competentes, os estudantes foram convidados a participar do estudo durante o horário de aula. Informou-se sobre a participação voluntária, destacando que a intervenção não causaria prejuízo quanto à dinâmica da escola e dos conteúdos a serem ministrados.

As etapas abaixo representam a dinâmica da coleta de dados:

Etapa I: Apresentação dos pesquisadores à equipe pedagógica; solicitação da frequência dos estudantes da EJA; realização da triagem inicial quanto à idade; apresentação dos pesquisadores em sala de aula; explicação sobre a pesquisa; e, após manifestação dos estudantes de seu interesse em participar, encaminhamento para espaço reservado pela escola para a coleta de dados.

Etapa II: Reafirmação do aceite para participar da pesquisa com assinatura do Termo de Consentimento Livre e Esclarecido (TCLE) em duas vias; aplicação do instrumento ilustrado (pré-teste) para avaliação do conhecimento basal, tanto no GI quanto no GC.

Etapa III: Intervenção Educacional.

Logo após o preenchimento do pré-teste, ocorreu a realização da intervenção educacional com o jogo “Mural de Risco”. O jogo em questão, desenvolvido por enfermeiras pesquisadoras na área da saúde sexual, possui um guia de uso que faz parte da composição do instrumento ilustrado para avaliar o conhecimento de idosos sobre prevenção de HIV/aids, ambos validados com Índice de Validade de Conteúdo (IVC = 0,90) e Escore *Suitability Assessment of Materials* de 22 pontos para o jogo e 24 pontos para o guia de uso ^(18)^. O jogo foi submetido também à avaliação do público-alvo, com obtenção do Índice de Concordância Semântico (ICS=0,80) ^(19)^. O guia explicativo traz informações importantes sobre a dinâmica do jogo, a saber: composição, finalidade, público-alvo, passo a passo do jogo e pontos reflexivos que podem ser abordados na discussão de cada imagem ao final da jogada.

O tabuleiro do jogo mede 1,00 x 1,00 metro, é articulado, contém 12 imagens coloridas, grandes, em vinil, com aplicação de Policloreto de Vinila (PVC) e com laminação transparente para proteção ^(18 , 20)^ (Figura 2).

**Figura 2 -:** Tabuleiro do jogo “Mural de Risco”. Recife, PE, Brasil, 2021

As imagens do jogo representam pessoas com 50 anos ou mais em diferentes situações cotidianas que podem indicar ou não risco para infecção pelo HIV. Acompanham o jogo 36 peças imantadas, sendo 6 na cor vermelha, 6 na cor amarela e 6 na cor verde, que representam muito risco, pouco risco ou nenhum risco para a infecção pelo HIV, respectivamente. O mediador organiza o tabuleiro e as peças, explica o objetivo do jogo e promove um momento de acolhimento e apresentação; o grupo elege um dos jogadores para ser o representante, todos veem atentamente o tabuleiro, analisam cada imagem e julgam se ela representa uma situação que indica muito, pouco ou nenhum risco de infecção pelo HIV.

A intervenção educacional ocorreu em grupos de 3 a 5 participantes, com duração média de trinta minutos, mediada por coletadores, previamente treinados para padronização do momento. Após o momento inicial de esclarecimentos sobre a dinâmica do jogo, os participantes foram dispostos em círculo, com o tabuleiro e as peças do jogo organizados em uma mesa central. Com a observação atenta do tabuleiro, os participantes analisaram as doze imagens, uma de cada vez, julgando, em consenso, o risco para a infecção pelo HIV, colocando nas figuras do mural a peça imantada vermelha, amarela ou verde. Quando todas as imagens estavam sobrepostas com uma peça imantada, a mediadora elevava o tabuleiro, transformando-o em um mural, permitindo que todos visualizassem as imagens. Ao final do jogo, a mediadora discutia sobre os acertos e erros, sanando possíveis dúvidas do grupo sobre a prevenção do HIV/aids.

Etapa IV: Aplicação do pós-teste imediato no GI após a jogada.

Etapa V: Reaplicação do instrumento pós-teste, após 30 dias, para análise do conhecimento tanto do GI quanto do GC.

### Tratamento e análise dos dados

Os dados coletados foram compilados no *Excel* e analisados por meio do *software* R. O nível de significância adotado foi de 5% e o intervalo de confiança de 95%. Utilizou-se a estatística descritiva para resumir um conjunto de observações. Para avaliar a normalidade dos dados contínuos e definir a escolha do teste (paramétrico ou não paramétrico) utilizou-se o teste Shapiro-Wilk. Para a comparação entre os grupos adotou-se os testes qui-quadrado e Fisher (variáveis categóricas) e o teste t de Student ou Mann-Whitney (variáveis contínuas). Para comparar a distribuição dos acertos, antes e depois da intervenção educacional, aplicou-se o teste não paramétrico de McNemar, que permitiu analisar as proporções entre as variáveis dicotômicas de forma pareada.

### Aspectos éticos

O estudo foi aprovado pelo Comitê de Ética em Pesquisa, sob o Parecer nº 4.258.634 e registrado na base de dados ReBEC: RBR-5w9tx9. Os participantes assinaram o TCLE, em duas vias, sendo assegurado seu anonimato, de acordo com as diretrizes da Resolução nº 466/12, do Conselho Nacional de Saúde ^(21)^.

## Resultados

A Figura 3 apresenta o número de estudantes, por escola, que compuseram a amostra do GI (n=50) e do GC (n=50).

A Tabela 1 apresenta a caracterização dos participantes.

**Figura 3 -:** Número de estudantes por escola. Recife, PE, Brasil, 2021

**Tabela 1 -:** Caracterização sociodemográfica dos estudantes (n* = 100). Recife, PE^†^, Brasil, 2021

**Características**	Geral (n* = 100)	**GC** ^‡^ (n* = 50)	**GI** ^§^ (n* = 50)	**p-valor** ^||^
**Sexo**				
Feminino	83 (83,0%)	43 (86,0%)	40 (80,0%)	0,594a ^¶^ (v** = 0,08)
Masculino	17 (17,0%)	7 (14%,0)	10 (20,0%)	
**Idade**				
Observações	100	50	50	0,025d ^††^ (r ^‡‡^ = 0,22)
Min-Máx ^§§^	50-82	50-82	50-74	
Q1-Q3 ^||||^	53,75-64	54,25-66	53,25-61	
Mediana	59	60	56	
Média	59,07	60,74	57,4	
Desvio-Padrão	6,86	7,42	5,85	
CV ^¶¶^	11,6%	12,2%	10,1%	
Normalidade (Shapiro-Wilk)	<0,001	0,052	0,008	
**Estado Civil**				
Casado(a)	34 (34,0%)	13 (26,0%)	21 (42,0%)	0,084a ^¶^ (v** = 0,29)
Divorciado(a)	12 (12,0%)	5 (10,0%)	7 (14,0%)	
Solteiro(a)	36 (36,0%)	18 (36,0%)	18 (36,0%)	
União Estável	2 (2,0%)	2 (4,0%)	0 (0,0%)	
Viúvo(a)	16 (16,0%)	12 (24,0%)	4 (8,0%)	
**Religião**				
Adventista	1 (1,0%)	1 (2,0%)	0 (0,0%)	0,895b***
Católica	44 (44,0%)	20 (40,0%)	24 (48,0%)	
Espírita	1 (1,0%)	1 (2,0%)	0 (0,0%)	
Evangélica	43 (43,0%)	22 (44%)	21 (42,0%)	
Afro-brasileira	1 (1,0%)	1 (2,0%)	0 (0,0%)	
Sem religião	10 (10,0%)	5 (10,0%)	5 (10,0%)	
**Escolaridade em anos de estudo**				
Observações	100	50	50	0,008d ^††^ (r ^‡‡^ = -0,27)
Min-Máx ^§§^	1-10	1-8	1-10	
Q1-Q3 ^||||^	1-4	1-3	2-5	
Mediana	2,5	2	3	
Média	3,03	2,5	3,56	
Desvio-Padrão	2,06	1,78	2,21	
CV ^¶¶^	68,0%	71,0%	61,9%	
Normalidade (Shapiro-Wilk)	<0,001	<0,001	<0,001	
**Vida sexual ativa**				
Não	55 (55,0%)	31 (62,0%)	24 (48,0%)	0,228a ^¶^ (v** = 0,14)
Sim	45 (45,0%)	19 (38,0%)	26 (52,0%)	

*n = Amostra; ^†^PE = Pernambuco; ^‡^GC = Grupo Controle; ^§^GI = Grupo Intervenção; ^||^p-valor = Nível de significância; ^¶^a = Teste Chi-Quadrado de independência; **v = v de Cramer; ^††^d = Teste de Mann-Whitney; ^‡‡^r = Estatística r; ^§§^Min-Máx = Mínimo e Máximo; ^||||^Q1-Q3 = Primeiro e terceiro quartis; ^¶¶^CV = Coeficiente de Variação; ***b = Teste Exato de Fisher

Em ambos os grupos houve predominância do sexo feminino. A escolaridade média no GC foi de 2,5 (±1,78) anos e no GI de 3,56 (±2,21) anos.

Os dois grupos se mostraram homogêneos, sendo que as características sociodemográficas dos participantes foram semelhantes, com diferença estatisticamente significativa apenas para a idade (p=0,025). Não foi identificada significância estatística (p=0,665) na comparação entre o conhecimento basal dos estudantes do GC (8,10) e GI (7,94), sobre prevenção do HIV/aids, evidenciando que os participantes, de ambos os grupos, detinham nível similar de conhecimento antes da exposição.

Entre o momento pré-intervenção e o período de 30 dias após a intervenção, o GI mostrou escores de conhecimento substancialmente superiores aos do GC, com diferença estatisticamente significativa (p<0,001). Enquanto o escore médio de conhecimento dos estudantes pertencentes ao GC foi de 8,06 (±2,24) pontos, o do GI foi de 10,26 (±1,43) (Tabela 2).

**Tabela 2 -:** Média dos escores de conhecimento de estudantes do GC* e do GI^†^ sobre prevenção do HIV/aids^‡^ imediatamente após a intervenção educacional. Recife, PE^§^, Brasil, 2021

**Grupos**					
**Período**	GC* (n=50)		GI ^†^ (n=50)		**p-valor** ^||^
	Média ± DP ^¶^	**IC** ^**^ **95%**	Média ± **DP** ^¶^	**IC** ^**^ **95%**	
Momento 0 (Basal)	8,10 ± ^¶^ 1,98	7,54 – 8,66	7,94 ± ^¶^ 2,04	7,36 – 8,52	0,665m ^††^
Imed. ^‡‡^ após	-	-	10,38 ± ^¶^ 1,28	10,02 – 10,74	-
Após 30 dias	8,06 ± ^¶^ 2,24	7,42 – 8,7	10,26 ± ^¶^ 1,43	9,85 – 10,67	<0,001m ^††^
Pré X Imed. ^‡‡^ após	-		<0,001w ^§§^		
Pré X Pós 30 dias	0,953w ^§§^		<0,001w ^§§^		

*GC = Grupo Controle; ^†^GI = Grupo Intervenção; ^‡^HIV/aids = Vírus da Imunodeficiência Humana; ^§^PE = Pernambuco; ^||^p-valor = Nível de significância; ^¶^DP = Desvio Padrão; **IC = Intervalo de Confiança; ^††^m = teste de Man Whitney; ^‡‡^Imed. = Imediatamente; ^§§^w = Wilcoxon

## Discussão

O aumento e a manutenção do conhecimento após 30 dias, evidenciados neste estudo, mostraram semelhança com resultados de outros estudos que, também, analisaram o conhecimento por meio do uso de jogos no processo de ensino-aprendizagem ^(22 - 23)^.

Este estudo gerou impacto para a prática clínica da enfermagem ao oferecer uma tecnologia validada e de baixo custo que poderá ser utilizada em ações educacionais em qualquer um dos três níveis de atenção à saúde com o intuito de prevenção de doenças ou agravos às pessoas com 50 anos ou mais.

Um dos cenários que as intervenções educacionais voltadas à educação sexual podem utilizar é o contexto escolar. Considerou-se para a escolha do público de 50 anos ou mais, estudantes da EJA, os altos índices de infecção pelo HIV nessa população; os conceitos equivocados dos profissionais de saúde e da educação a respeito da sexualidade para esse grupo; a deficiência na formação pedagógica dos docentes da EJA para a abordagem das temáticas sobre saúde sexual; a fragilidade quanto à composição curricular; e o debate e atenção da saúde sexual, ainda muito precários no ambiente escolar ^(24 - 25)^. Ademais, o aumento na idade implica em maior risco de doenças e condições relacionadas à saúde, portanto, indivíduos com 50 anos ou mais apresentam progressão para a imunossupressão de forma mais acelerada, observando-se, ainda, maior aparecimento de doenças oportunistas, acrescido de maior probabilidade do advento de outras comorbidades, o que contribui para o agravamento das condições de saúde ^(26)^.

Embora haja barreiras para trabalhar a importância da prevenção do HIV/aids com a população em questão, a exemplo do comportamento sexual de risco, do conhecimento precário sobre sexualidade e das dificuldades ou impossibilidades de engravidarem, a intervenção educacional, por meio do jogo “Mural de Risco”, mostrou-se efetiva para o aumento do conhecimento no momento basal e após a intervenção sobre a prevenção do HIV/aids em pessoas com 50 anos ou mais em contexto escolar.

No entanto, esse resultado, ao passo que demonstra uma evidência positiva, também suscita uma reflexão quanto à importância de realizar investimentos em ações de educação em saúde que estimulem a prática sexual segura ^(27)^, uma vez que o acesso aos conhecimentos acerca da prevenção do HIV/aids, por si só, pode não ser suficiente ou simplesmente não implicar eficazmente em mudança das práticas sexuais desprotegidas.

No que concerne à religiosidade, quase metade dos participantes eram católicos. Ao avaliar a espiritualidade, a religiosidade e as crenças do público em questão, constatou-se, em estudo similar, que os aspectos espirituais e religiosos estão comumente presentes na vida dessas pessoas. O fato de, comumente, a religião associar a sexualidade somente ao ato sexual em si e não à compreensão desta em sua totalidade, pode ser um fator inibitório para a prática sexual por parte deste público ou, até mesmo, um fator de risco por poder gerar um sentimento de repulsa ^(28)^.

Percebeu-se, durante a realização do estudo, que para além de testar a efetividade do jogo, evidenciou-se também a importância da realização de ações de educação em saúde sobre temáticas alusivas à sexualidade com o público em questão, com a utilização de tecnologias educacionais, uma vez que estas são capazes de estimular, sobremaneira, a cognição por meio do uso da visão, audição e tato, o que impacta diretamente na compreensão da temática e na motivação pelo aprendizado. Destaca-se ainda que estas ações devem ser estimuladas e realizadas pelas equipes na educação básica.

Quanto aos anos de estudo, dado semelhante verificado em investigação sobre perfil socioepidemiológico e autonomia de longevos ^(29)^, demonstrou que 46,0% do público apresentou entre um e quatro anos de estudo. Outra pesquisa, realizada no norte do Brasil ^(28)^ sobre alfabetização em saúde acerca do HIV, constatou que a maioria das pessoas não tinha escolaridade ou havia concluído apenas o nível fundamental. Observa-se que o baixo nível de alfabetização favorece o aumento da vulnerabilidade à infecção pelo HIV e, portanto, há que se reunir esforços para dar mais atenção e visibilidade ao cuidado voltado à saúde sexual deste público.

A inatividade sexual revelada pelo público-alvo deste estudo é uma realidade que corrobora aquela identificada em pesquisa que mostrou que fatores como a presença de comorbidades, as mudanças corporais, a impotência sexual e a visão social deturpada da sexualidade fazem com que pessoas com 50 anos ou mais não se sintam à vontade ou até mesmo sintam-se culpadas ao expressar seus desejos sexuais ^(30)^. No entanto, durante as jogadas, os estudantes verbalizaram a vontade e o interesse sexual.

Dada a exposição física, cultural e sexual ao HIV, constata-se a relevância de, diante dos dados de perfil sociodemográficos aqui apresentados, clarificar aspectos importantes que auxiliem em uma melhor atenção à saúde sexual da pessoa idosa. Tais achados podem contribuir para o cuidado de enfermagem, por meio do melhor planejamento e direcionamento das orientações, diálogos, condutas e do letramento em saúde nessa população. Destarte, há que se ofertar um espaço inclusivo e direcionado, com escuta qualificada, diálogo livre de julgamentos, estabelecimento de vínculos e práticas baseadas em evidências ^(31)^.

Uma revisão sistemática de ensaios clínicos randomizados ^(32)^, sobre jogos e abordagens gamificadas na área da saúde, evidenciou que a maioria dos estudos primários resultou no aumento significativo de conhecimento por meio de jogos de tabuleiro em relação a outros jogos. A metanálise realizada nessa revisão mostrou um efeito médio dos jogos de tabuleiro no conhecimento relacionado à saúde (d* = 0,82, intervalo de confiança de 95%; IC [0,15–1,48]), com aumento do conhecimento sobre saúde da maioria das intervenções que utilizou jogos de tabuleiro (76,0%, n = 16) ^(32)^.

Ao garantir a efetividade do “Mural de Risco” contribui-se para o corpo de conhecimento da enfermagem baseada em evidências com a inclusão do paciente no processo do cuidado, considerado um desafio ^(33 - 34)^. A tecnologia mostrou-se, portanto, útil na disseminação do conhecimento sobre a prevenção do HIV ao tempo que favoreceu o protagonismo no autocuidado e, com isso, acredita-se que tem potencial de contribuir com a diminuição dos índices de HIV/aids no público em questão.

O estudo poderá, portanto, contribuir para o cuidado de enfermagem planejado e direcionado quanto às orientações, diálogos e condutas em saúde para essa população. Com o uso do “Mural de Risco”, tecnologia de baixo custo e fácil acesso, pelos enfermeiros durante as ações de educação em saúde, será possível ofertar um espaço inclusivo e direcionado ao público com 50 anos ou mais, com escuta qualificada, diálogo livre de julgamentos, estabelecimento de vínculos e práticas baseadas em evidências.

Diante do exposto, e tendo em vista os resultados que os jogos de tabuleiro têm no conhecimento direcionado à saúde, a exemplo do conhecimento sobre HIV e IST, além de suas implicações para o avanço do conhecimento científico para a área de saúde e da enfermagem, sugere-se que os resultados aqui discutidos não se esgotam neste estudo.

Considera-se como limitações do estudo o potencial viés de cortesia, com aplicação dos instrumentos realizada antes e após um momento lúdico; o fato de não terem sido consideradas, para a análise dos dados, as divergências sociais, relacionais e emocionais das fases de vida dos participantes; e a avaliação da retenção de conhecimento após a intervenção proposta realizada apenas de forma imediata e a médio prazo. Sugere-se, assim, que novos estudos de caráter analítico e/ou qualitativo sejam desenvolvidos para avaliar o fenômeno em questão.

Destaca-se que o público-alvo do estudo foi ampliado para a faixa etária de 50 anos ou mais, diferente do registro no protocolo ReBEC que considerou a inclusão de pessoas a partir de 60 anos, em razão do perfil epidemiológico da atualidade que evidencia elevados índices do HIV nessa população específica ^(1)^.

## Conclusão

O jogo de tabuleiro “Mural de Risco” foi efetivo no aumento do conhecimento das pessoas com 50 anos ou mais que participaram das intervenções educacionais. Pode ser utilizado, portanto, na assistência de enfermagem às pessoas com 50 anos ou mais, seja no contexto escolar ou nos níveis de atenção à saúde, de forma a possibilitar que essa população conheça as formas de prevenção do Vírus da Imunodeficiência Adquirida/aids.

No intuito de identificar lacunas de pesquisa que porventura não tenham sido atendidas, recomenda-se a realização de novas investigações que envolvam a temática em questão e o uso deste jogo em outros contextos, a exemplo de pessoas com 50 anos ou mais que possuam alta escolaridade.

Ressalta-se, por fim, que o jogo “Mural de Risco”, tecnologia educacional registrada na Biblioteca Nacional (nº de registro: 878.773 - livro: 1712 - folha: 481), pode ser replicado com baixo custo, inclusive de forma artesanal, tornando o aprendizado sobre a prevenção do HIV/aids mais acessível e democrático.

# Translation: Efectividad de la tecnología educativa sobre prevención del virus de la inmunodeficiencia humana/sida: ensayo clínico aleatorizado*

**DOI:** 10.1590/1518-8345.7352.4514

**Authors:** Priscila Cabral Melo Holanda, Wilson Jorge Correia de Abreu, Francisca Márcia Pereira Linhares, Ryanne Carolynne Marques Gomes Mendes, Fábia Alexandra Pottes Alves, Tatiane Gomes Guedes

**Affiliations:** 1 Universidade Federal de Pernambuco, Departamento de Enfermagem, Recife, PE, Brasil.; 2 Becaria de la Coordenação de Aperfeiçoamento de Pessoal de Nível Superior (CAPES), Brasil.; 3 Escola Superior de Enfermagem do Porto, Enfermagem, Porto, Portugal.

**Keywords:** Tecnología Educacional, Enfermería, Anciano, VIH, Prevención de Enfermedades, Síndrome de Inmunodeficiencia Adquirida

## Abstract

evaluar la efectividad del juego de mesa *Mural de Risco* sobre prevención del Virus de Inmunodeficiencia Humana/sida en el conocimiento de personas de 50 años o más en contexto escolar.

ensayo controlado aleatorizado, con dos brazos, realizado en 18 colegios con 100 personas de Educación de Jóvenes y Adultos. Para evaluar los conocimientos se utilizó un instrumento ilustrado validado. La intervención educativa se desarrolló en grupos de 3 a 5 participantes, utilizando el juego *Mural de Risco*, en la que los participantes evaluaban las imágenes que representaban mucho, poco o ningún riesgo de infección por VIH. En la distribución de respuestas correctas se aplicó la prueba de McNemar.

se observó un aumento significativo en los puntajes medios del grupo intervención entre el momento inicial y el trigésimo día (p=0,001), no ocurrió lo mismo en el grupo control (p=0,953).

el juego fue efectivo para aumentar el conocimiento de las personas de 50 años o más sobre la prevención del Virus de Inmunodeficiencia Humana/sida. Registro Brasileño de Ensayos Clínicos: RBR-5w9tx9.

## Destacados

**(1)** En Brasil y en el mundo los adultos mayores tienen altas tasas de VIH/sida. **(2)** El juego de mesa es un recurso educativo de bajo costo y fácil acceso. **(3)** El juego de mesa contiene imágenes de adultos mayores en situaciones cotidianas. **(4)** El juego *Mural de Risco* fue validado por expertos y evaluado por el público objetivo. **(5)** El juego puede ser utilizado por enfermeros en acciones de educación para la salud.

## Introducción

El aumento del número de casos del Síndrome de Inmunodeficiencia Adquirida (sida) intensifica los efectos de las desigualdades sociales y afecta principalmente a las personas en situación de vulnerabilidad. A nivel mundial, millones de vidas todavía están en riesgo y son afectadas por el Virus de Inmunodeficiencia Humana (VIH). En el Boletín Epidemiológico VIH/sida 2023 se puede identificar que hubo un aumento del número de nuevos registros de VIH en personas de 50 años o más, al comparar los periodos de 2012 a 2017 y de 2018 a 2023, de 21.861 a 29.821 casos, respectivamente ^(1)^. Una de las estrategias del *Joint United Nations Programme on* HIV/aids - UNAIDS ^(2)^ para reducir los costos de la atención en salud a largo plazo es incluir a ese público específico en las acciones de lucha contra el VIH/sida.

El UNAIDS también destaca que los datos sobre la respuesta global al VIH para 2025 son preocupantes: las nuevas infecciones podrían alcanzar los 1,2 millones. Se reitera que durante los últimos dos años -periodo en el que ocurrieron la pandemia provocada por el nuevo coronavirus y otras crisis globales- se han debilitado los avances contra la pandemia del VIH y hubo una disminución de los recursos y, por lo tanto, millones de vidas están en riesgo. Europa del Este, Asia Central, Oriente Medio, África del Norte y América Latina están experimentando aumentos significativos en las tasas anuales de infección. A nivel mundial, la tasa de nuevas infecciones disminuyó solo un 3,6% entre 2020 y 2021 ^(2)^.

En todas las regiones de Brasil, la incidencia del VIH/sida ha aumentado en las personas de 50 años o más. En 2022, Pernambuco registró el segundo mayor número de casos de VIH de la región Nordeste ^(1)^.

Son muchos los factores que exponen al citado grupo, como la inmunosenescencia, que comienza a manifestarse de diferentes formas, de manera paulatina y continua, generalmente a partir de los 50 años; el uso inadecuado del preservativo; la falta de apoyo emocional; el bajo nivel educativo; la escasa discusión sobre la sexualidad en el envejecimiento, que conduce a una falta de conocimiento sobre la prevención del VIH; entre otros. Por ende, es importante fomentar en esta población el uso de la Profilaxis Pre-Exposición (PrEP) y la Profilaxis Post-Exposición (PEP) ^(3)^. Además, es necesario evaluar el estilo de vida, las condiciones socioeconómicas y los diferentes factores que pueden intervenir en las infecciones por VIH ^(4)^.

Otro factor determinante en la atención a este público objetivo es considerar no sólo la edad cronológica, sino, sobre todo, la concepción de la edad biológica. Desde esta perspectiva, los estudios que incluyen la prevención del VIH y las personas de 50 años o más están ganando cada vez más importancia y protagonismo, ya que contribuyen al conocimiento sobre la adherencia a conductas sexuales seguras y, por ende, a reducir las Enfermedades de Transmisión Sexual (ETS) ^(5)^.

No incluir a las personas de 50 años o más en los planes estratégicos y las políticas públicas orientadas a la salud sexual de adultos de mediana edad y adultos mayores las hace aún más vulnerables y tiene un impacto directo sobre la visibilidad de las mismas con respecto a la relación exposición/prevención del VIH. Esta vulnerabilidad puede estar asociada a las relaciones sexuales sin protección, al uso de medicamentos para la disfunción eréctil y a la invisibilidad de la sexualidad de las personas de 50 años o más. En cuanto a la prevención del VIH/sida para la población de adultos mayores en Brasil, solo hay una directriz, de 2017, que menciona a las personas mayores y se relaciona con las particularidades de esa población para implementar la red de atención ^(6)^.

El panorama epidemiológico y la realidad social de dicho público frente al VIH plantean que es necesario que se realicen cambios en la concepción social y biológica sobre la infección por VIH/sida, y al mismo tiempo indican lo importante que es invertir en acciones de prevención de forma cada vez más temprana ^(7)^.

En este contexto, hay que destacar a los profesionales de enfermería y cómo contribuyen, a nivel local y global frente al VIH/sida, con medidas clínico-asistenciales y comportamentales, al brindar atención dirigida a luchar contra el VIH, mediante acciones centradas en la escucha calificada ^(8)^ y en el desarrollo de tecnologías educativas ^(9)^.

La tecnología educativa, herramienta elaborada en base al conocimiento científico, puede ser utilizada como material en el escenario educativo, como por ejemplo el juego de mesa. Un estudio que utilizó esta herramienta en acciones de salud identificó que contribuyó a la adopción de cambios de comportamiento, tales como llevar una alimentación saludable, dejar de fumar y tener sexo seguro, y a que se obtuvieran mejoras en los casos de deterioro cognitivo y de depresión ^(10)^.

En el ámbito educativo, la escuela se presenta como una institución indispensable para la educación de las personas, para colaborar con la adquisición de conocimiento que promueva conductas saludables, como la prevención del VIH/sida. Por lo tanto, es un espacio para que los enfermeros actúen para llegar al público adulto y adulto mayor a través de la Educación de Jóvenes y Adultos (EJA), del Ministerio de Educación de Brasil. Esta acción está anclada en el Programa de Salud Escolar (PSE), una estrategia de integración de salud y educación para el desarrollo de la ciudadanía, del Ministerio de Salud de Brasil ^(11)^.

Por consiguiente, es importante realizar estudios que prueben la efectividad de los juegos de mesa, centrados en la adquisición de conocimiento seguro, dirigidos a personas de 50 años o más, acordes al perfil epidemiológico del VIH de este grupo específico, que se sumen a la tendencia de los estudios centrados en la lucha contra el VIH en la población joven, sexualmente activa o con prácticas sexuales de riesgo. Los juegos de mesa, en este sentido, pueden innovar, de forma sencilla y lúdica, la educación para la prevención del VIH en personas de 50 años o más. También pueden proporcionar experiencias interactivas que se acerquen a la realidad de este público y, así, desmitificar tabúes relacionados con la prevención del VIH, que aún están muy presentes en la sociedad.

Para reforzar la utilidad y relevancia sociocientífica de las tecnologías educativas, el objetivo es evaluar la efectividad del juego de mesa *Mural de Risco* (Mural de Riesgo) en el conocimiento de personas de 50 años o más, en el contexto escolar, sobre la prevención del VIH/sida.

## Método

### Diseño del estudio

Este es un ensayo clínico controlado aleatorizado no farmacológico, con dos brazos: intervención y control. Se siguieron las directrices de los *Consolidated Standards of Reporting Trials* (CONSORT) ^(12)^ para grupos paralelos y el estudio fue registrado en el Registro Brasileño de Ensayos Clínicos (ReBEC): RBR-5w9tx9.

### Lugar

Las 18 escuelas (estatales y municipales) en Recife (PE), Brasil, en las que se realizó el presente estudio funcionaban en horario nocturno y daban cuatro horas de clases por día. La EJA, en la ciudad de Recife, tiene más de 25 mil estudiantes matriculados en el turno nocturno que cursan la educación primaria, la misma está dividida en cinco módulos y tiene una duración media de cinco años.

### Período

La investigación se desarrolló durante los meses de octubre y noviembre de 2021.

### Población

Se eligió a personas de 50 años o más, incluidos los adultos mayores, para conformar este estudio debido al importante crecimiento del número de casos de SIDA en esta población, al descuido en la inclusión de estas personas en las políticas de prevención del VIH/sida y a la inmunosenescencia presente en dicha franja etaria ^(13)^. La población estuvo compuesta por 100 personas de 50 años o más, asignadas 50 al Grupo intervención (GI) y 50 al Grupo Control (GC). La aleatorización del Estudio Aleatorizado Controlado se llevó a cabo por conglomerados, las personas fueron asignadas en grupos (escuelas) y no individualmente ^(14)^.

### Criterios de selección

Los criterios de inclusión fueron: ser personas de 50 años o más, estar matriculados regularmente en colegios estatales o municipales que tuvieran la modalidad EJA y tener al menos seis meses de estudio en esta modalidad. Se excluyeron las personas de 50 años o más que no asistieron a clases durante el período de recolección de datos. El criterio de discontinuidad fue personas de 50 años o más que abandonaron los estudios (deserción escolar) después del inicio de la recolección.

### Definición de la muestra

Para determinar el tamaño de la muestra se utilizó la ecuación de cálculo de muestra para dos proporciones experimentales ^(15)^, nivel de confianza del 95%, poder de la prueba del 90% y con la prevalencia esperada de conocimiento y desconocimiento sobre la prevención de la enfermedad VIH/sida, se obtuvo un tamaño de muestra de 45 personas de 50 años o más para cada grupo. Al considerar una posible pérdida del 10% (cinco personas de 50 años o más), el número necesario en cada grupo era de 50 observaciones, es decir, 100 participantes en la muestra.

La muestra se determinó por conveniencia, según la disponibilidad de los estudiantes para la recolección de datos en los lugares de estudio. Inicialmente se identificó que 215 estudiantes cumplían con los criterios establecidos. Sin embargo, 68 no estaban asistiendo a clases durante el período de recolección de datos. Por lo tanto, 147 personas eran elegibles, de las cuales ocho se negaron a participar. Entonces, se asignaron 139, 74 al GI y 65 al GC. Después de la pérdida sufrida durante el seguimiento (ausencia durante el período de recolección), 50 estudiantes permanecieron en el GI y 50 estudiantes en el GC (Figura 1).

A pesar de la similitud de la muestra, estudiantes de la EJA de 50 años o más, la adquisición de conocimiento por parte de los participantes durante el período de recolección de datos sobre la prevención del VIH/sida puede ser considerada un potencial factor de confusión en el estudio.

**Figura 1 -:** Diagrama de flujo de la recolección de datos según *Consolidated Standards of Reporting Trials* ^(12)^. Recife, PE, Brasil, 2021

### Variables del estudio

Variables independientes, se evaluaron variables sociodemográficas: género, edad, estado civil, religión, educación (años de estudio) y hábitos de vida relacionados con la salud sexual (cómo considera la salud, vida sexual activa y actividad física).

Variables dependientes: se consideró como variable dependiente el conocimiento sobre la prevención del VIH/sida, medido a través del puntaje obtenido en la evaluación que se realizó durante la entrevista inicial y, posteriormente, al trigésimo día, tanto en el GI como en el GC.

### Instrumento de recolección de datos

Para la recolección de datos se utilizó un instrumento ilustrado, validado por expertos en salud y evaluado por el público objetivo. El instrumento consta de 12 preguntas cortas de opción múltiple cuyas opciones son: “verdadero”, “falso” y “no sé” ^(16)^. Las preguntas involucran situaciones cotidianas que experimentan las personas de 50 años o más en un contexto de riesgo de infección por VIH, como: hacer gimnasia acuática, viajar en autobús, toser, abrazarse, utilizar objetos punzocortantes, entre otras ^(16)^.

Las ilustraciones del instrumento provienen del tablero *Mural de Risco*, desarrollado por los diseñadores del juego. El resultado del mencionado instrumento ilustrado debe ser analizado cuantitativamente, a saber: nueve o más preguntas correctas indican que tienen un conocimiento adecuado sobre la enfermedad y diferentes resultados revelan conocimiento inadecuado sobre la infección por VIH y sida. Cabe aclarar que la respuesta “no sé” se clasifica como incorrecta, es decir, demuestra conocimiento insuficiente sobre el tema en cuestión ^(16)^.

### Recolección de datos

Para evaluar la efectividad del juego de mesa *Mural de Risco* en el conocimiento de personas de 50 años o más en un contexto escolar sobre la prevención del VIH/sida, las personas fueron asignadas de manera aleatoria y uniforme, al GI y al GC. Para evitar la contaminación de los participantes del grupo, hecho que podría ocurrir si la aleatorización fuera realizada individualmente, dado que los estudiantes frecuentan el mismo establecimiento educativo, el tipo de aleatorización adoptado en este estudio fue por conglomerado o *cluster* ^(17)^.

Antes de contactar a los estudiantes, se formaron los conglomerados. Esto se realizó mediante un muestreo aleatorio simple para definir las escuelas que formarían parte de los conglomerados A (GI) y B (GC). Para garantizar el secreto de la asignación, se utilizó un sobre cerrado y opaco con los nombres de las escuelas y el respectivo número de estudiantes. Para componer cada conglomerado se realizó un sorteo: primero de las escuelas del grupo A - GI, y luego de las escuelas del grupo B - GC, de modo que cada grupo tuviera aproximadamente el mismo número de estudiantes. Según el cálculo de la muestra, en cada grupo se contabilizó un número de estudiantes que, después de predecir las pérdidas, fuera al menos de 45 en cada grupo. Este tipo de aleatorización por conglomerados o *clusters* adapta mejor las preguntas de investigación sobre los programas de salud y sus efectos en la población ^(17)^.

La intervención educativa con el mencionado juego de mesa, así como la aplicación de la evaluación previa y la posterior, fueron realizadas por un equipo de 12 investigadores previamente capacitados y calibrados, a través de un estudio piloto. Al principio se realizó una evaluación inicial, mediante la aplicación del pretest en el GI y GC, seguida de una segunda evaluación en la que se aplicó el postest inmediatamente después de la intervención educativa en el GI y, además, a los 30 días posteriores a la entrevista inicial se aplicó el postest en ambos grupos, para evaluar la retención de conocimiento de los participantes del estudio durante este período.

Cabe destacar que los puntajes de conocimiento del GC se midieron en base a la exposición natural de este grupo (conocimiento adquirido habitualmente en el día a día).

Con el consentimiento previo de las autoridades escolares competentes, los estudiantes fueron invitados a participar del estudio durante el horario de clase. Se les informó que la participación era voluntaria y se destacó que la intervención no afectaría la dinámica de la escuela ni los contenidos que se iban a impartir.

Las siguientes etapas representan la dinámica de la recolección de datos:

Etapa I: Presentación de los investigadores al equipo pedagógico; solicitud de la asistencia de los estudiantes de la EJA; realización del cribado inicial por edad; presentación de los investigadores en el aula; explicación sobre la investigación; y, luego de que los estudiantes expresaron su interés en participar, conducción de los mismos a un espacio reservado por la escuela para la recolección de datos.

Etapa II: Reafirmación de la aceptación para participar en la investigación mediante la firma de dos copias del Formulario de Consentimiento Libre e Informado (FCLI); aplicación del instrumento ilustrado (pretest) para evaluar el conocimiento inicial, tanto en el GI como en el CG.

Etapa III: Intervención Educativa.

Después de finalizar el pretest se llevó a cabo la intervención educativa con el juego *Mural de Risco*. El juego en cuestión, desarrollado por enfermeras investigadoras en el área de salud sexual, cuenta con un manual de uso que forma parte de la composición del instrumento ilustrado para evaluar el conocimiento de las personas mayores sobre la prevención del VIH/SIDA, ambos validados con un Índice de Validez de Contenido (IVC = 0,90) y Puntaje de *Suitability Assessment of Materials* de 22 puntos para el juego y 24 puntos para el manual de uso ^(18)^. El juego también fue sometido a la evaluación del público objetivo y obtuvo un Índice de Concordancia Semántica de (ICS=0,80) ^(19)^. El manual explicativo brinda información importante sobre la dinámica del juego, a saber: composición, propósito, público objetivo, paso a paso del juego y puntos de reflexión que se pueden abordar en la discusión de cada imagen al final de cada jugada.

El tablero del juego mide 1,00 x 1,00 metro, es articulado, contiene 12 imágenes grandes y a color, en vinilo, con aplicación de Policloruro de Vinilo (PVC) y una lámina transparente para protección ^(18 , 20)^ (Figura 2).

**Figura 2 -:** Tablero del juego *Mural de Risco*. Recife, PE, Brasil, 2021

Las imágenes del juego representan a personas de 50 años o más en diferentes situaciones cotidianas que pueden o no indicar riesgo de infección por VIH. El juego viene con 36 piezas imantadas, 6 rojas, 6 amarillas y 6 verdes, que representan mucho riesgo, poco riesgo o ningún riesgo de infección por VIH, respectivamente. El mediador organiza el tablero y las piezas, explica el objetivo del juego y promueve un momento de bienvenida y presentación; el grupo elige a uno de los jugadores como representante, todos miran atentamente el tablero, analizan cada imagen y evalúan si representa una situación que indica mucho, poco o ningún riesgo de infección por VIH.

La intervención educativa se desarrolló en grupos de 3 a 5 participantes, con una duración promedio de treinta minutos, mediada por recolectores, previamente capacitados para estandarizar el momento. Luego del momento inicial en el que se aclaró cómo sería la dinámica del juego, los participantes fueron ubicados en círculo, con el tablero y las piezas del juego organizados en una mesa central. Observando atentamente el tablero, los participantes analizaron las doce imágenes, una por una, evaluaron, por consenso, el riesgo de infección por VIH y colocaron la pieza magnética roja, amarilla o verde sobre las figuras del mural. Una vez que se le había asignado una pieza de imán a cada una de las imágenes, la mediadora levantó el tablero transformándolo en un mural, y permitió que todos pudieran visualizar las imágenes. Al final del juego, la mediadora discutió los aciertos y errores, y aclaró posibles dudas que tenía el grupo sobre la prevención del VIH/sida.

Etapa IV: Aplicación del postest inmediatamente después del juego en el GI.

Etapa V: Reaplicación del instrumento postest, después de 30 días, para analizar el conocimiento tanto del GI como del GC.

### Procesamiento y análisis de los datos

Los datos recolectados fueron compilados en *Excel* y analizados mediante el *software* R. El nivel de significación adoptado fue del 5% y el intervalo de confianza del 95%. Se utilizó estadística descriptiva para resumir un conjunto de observaciones. Para evaluar la normalidad de los datos continuos y definir la elección de la prueba (paramétrica o no paramétrica) se utilizó la prueba de Shapiro-Wilk. Para comparar los grupos se utilizaron las pruebas de chi-cuadrado y de Fisher (variables categóricas) y la prueba t de Student o de Mann-Whitney (variables continuas). Para comparar la distribución de respuestas correctas, antes y después de la intervención educativa, se aplicó la prueba no paramétrica de McNemar, que permitió analizar las proporciones entre las variables dicotómicas de forma pareada.

### Aspectos éticos

El estudio fue aprobado por el Comité de Ética en Investigación, bajo dictamen N° 4.258.634 y registrado en la base de datos del ReBEC: RBR-5w9tx9. Los participantes firmaron el FCLI, dos copias, y se garantizó el anonimato de los mismos, según las directrices de la Resolución N° 466/12, del Consejo Nacional de Salud ^(21)^.

## Resultados

La Figura 3 presenta el número de estudiantes, por escuela, que conformaron la muestra del GI (n=50) y GC (n=50).

La Tabla 1 presenta la caracterización de los participantes.

**Figura 3 -:** Número de estudiantes por escuela. Recife, PE, Brasil, 2021

**Tabla 1 -:** Caracterización sociodemográfica de los estudiantes (n* = 100). Recife, PE^†^, Brasil, 2021

**Características**	General (n* = 100)	**GC** ^‡^ (n* = 50)	**GI** ^§^ (n* = 50)	**valor p** ^||^
**Sexo**				
Femenino	83 (83,0%)	43 (86,0%)	40 (80,0%)	0,594a ^¶^ (v** = 0,08)
Masculino	17 (17,0%)	7 (14%,0)	10 (20,0%)	
**Edad**				
Observaciones	100	50	50	0,025d ^††^ (r ^‡‡^ = 0,22)
Min-Máx ^§§^	50-82	50-82	50-74	
Q1-Q3 ^||||^	53,75-64	54,25-66	53,25-61	
Mediana	59	60	56	
Media	59,07	60,74	57,4	
Desviación Estándar	6,86	7,42	5,85	
CV ^¶¶^	11,6%	12,2%	10,1%	
Normalidad (Shapiro-Wilk)	<0,001	0,052	0,008	
**Estado Civil**				
Casado(a)	34 (34,0%)	13 (26,0%)	21 (42,0%)	0,084a ^¶^ (v** = 0,29)
Divorciado(a)	12 (12,0%)	5 (10,0%)	7 (14,0%)	
Soltero(a)	36 (36,0%)	18 (36,0%)	18 (36,0%)	
Unión Estable	2 (2,0%)	2 (4,0%)	0 (0,0%)	
Viudo(a)	16 (16,0%)	12 (24,0%)	4 (8,0%)	
**Religi** ón				
Adventista	1 (1,0%)	1 (2,0%)	0 (0,0%)	0,895b***
Católica	44 (44,0%)	20 (40,0%)	24 (48,0%)	
Espiritista	1 (1,0%)	1 (2,0%)	0 (0,0%)	
Evangélica	43 (43,0%)	22 (44%)	21 (42,0%)	
Afrobrasileña	1 (1,0%)	1 (2,0%)	0 (0,0%)	
Sin religión	10 (10,0%)	5 (10,0%)	5 (10,0%)	
**Educación en años de estudio**				
Observaciones	100	50	50	0,008d ^††^ (r ^‡‡^ = -0,27)
Min-Máx ^§§^	1-10	1-8	1-10	
Q1-Q3 ^||||^	1-4	1-3	2-5	
Mediana	2,5	2	3	
Media	3,03	2,5	3,56	
Desviación Estándar	2,06	1,78	2,21	
CV ^¶¶^	68,0%	71,0%	61,9%	
Normalidad (Shapiro-Wilk)	<0,001	<0,001	<0,001	
**Vida sexual activa**				
No	55 (55,0%)	31 (62,0%)	24 (48,0%)	0,228a ^¶^ (v** = 0,14)
Sí	45 (45,0%)	19 (38,0%)	26 (52,0%)	

*n = Muestra; ^†^PE = Pernambuco; ^‡^GC = Grupo Control; ^§^GI = Grupo Intervención; ^||^valor p = Nivel de significación; ^¶^a = Prueba chi-cuadrado de independencia; **v = v de Cramer; ^††^d = Teste de Mann-Whitney; ^‡‡^r = Estadística r; ^§§^Min-Máx = Mínimo y Máximo; ^||||^Q1-Q3 = Primer y tercer cuartil; ^¶¶^CV = Coeficiente de Variación; ***b = Prueba Exacta de Fisher

En ambos grupos predominó el sexo femenino. La educación promedio en el grupo control fue de 2,5 (±1,78) años y en el grupo intervención de 3,56 (±2,21) años.

Los dos grupos fueron homogéneos y las características sociodemográficas de los participantes fueron similares, sólo hubo diferencia estadísticamente significativa en la edad (p=0,025). No se identificó significación estadística (p=0,665) al comparar el conocimiento inicial de los estudiantes del GC (8,10) y del GI (7,94) sobre la prevención del VIH/sida, lo que demuestra que los participantes, de ambos grupos, poseían un nivel similar de conocimiento antes de la exposición.

Entre el momento previo a la intervención y los 30 días posteriores a la intervención, el GI obtuvo puntajes de conocimiento sustancialmente mayores que el GC, con diferencia estadísticamente significativa (p<0,001). Mientras que el puntaje promedio del conocimiento de los estudiantes pertenecientes al GC fue de 8,06 (±2,24) puntos, el del GI fue de 10,26 (±1,43) (Tabla 2).

**Tabla 2 -:** Puntajes promedio de conocimiento de los estudiantes de CG* y GI^†^ sobre la prevención del VIH/sida^‡^ inmediatamente después de la intervención educativa. Recife, PE^§^, Brasil, 2021

**Grupos**					
**Periodo**	GC* (n=50)		GI ^†^ (n=50)		**valor p** ^||^
	**Media ± DE** ^¶^	**IC** ^**^ **95%**	**Media ±** **DE** ^¶^	**IC** ^**^ **95%**	
Momento 0 (Inicial)	8,10 ± ^¶^ 1,98	7,54 – 8,66	7,94 ± ^¶^ 2,04	7,36 – 8,52	0,665m ^††^
Inmed. ^‡‡^ después	-	-	10,38 ± ^¶^ 1,28	10,02 – 10,74	-
Después de 30 días	8,06 ± ^¶^ 2,24	7,42 – 8,7	10,26 ± ^¶^ 1,43	9,85 – 10,67	<0,001m ^††^
Pre vs. Inmed. ^‡‡^ después	-		<0,001w ^§§^		
Pre vs. Post 30 dias	0,953w ^§§^		<0,001w ^§§^		

*GC = Grupo Control; ^†^GI = Grupo Intervención; ^‡^VIH/sida = Virus de Inmunodeficiencia Humana; ^§^PE = Pernambuco; ^||^valor p = Nivel de significación; ^¶^DE = Desviación Estándar; **IC = Intervalo de Confianza; ^††^m = prueba de Man Whitney; ^‡‡^Inmed. = Inmediatamente; ^§§^w = Wilcoxon

## Discusión

El aumento y mantenimiento del conocimiento después de 30 días, observados en este estudio, son similares a los presentes en los resultados de otros estudios que también analizaron el conocimiento mediante el uso de juegos en el proceso de enseñanza-aprendizaje ^(22 - 23)^.

Este estudio tuvo un impacto en la práctica clínica de enfermería al proporcionar una tecnología validada y de bajo costo que puede ser utilizada en acciones educativas en cualquiera de los tres niveles de atención de la salud con el objetivo de prevenir enfermedades o problemas de salud en personas de 50 años o más.

Uno de los escenarios que pueden utilizar las intervenciones educativas dirigidas a la educación sexual es el contexto escolar. Cuando se eligió como público personas de 50 años o más, estudiantes de la EJA, se consideraron los altos índices de infección por VIH en dicha población; las concepciones erróneas de los profesionales de la salud y de la educación sobre la sexualidad para ese grupo; la escasa formación pedagógica de los docentes de la EJA para abordar temas de salud sexual; las falencias en el contenido curricular; y el debate y la atención para la salud sexual, aún muy precario en el ámbito escolar ^(24 - 25)^. Además, el aumento de la edad implica un mayor riesgo de padecer enfermedades y afecciones relacionadas con la salud, por lo que los individuos de 50 años o más presentan una progresión más acelerada hacia la inmunosupresión, por lo que se observa mayor aparición de enfermedades oportunistas, además de mayor probabilidad de que surjan otras comorbilidades, que contribuyen a empeorar las condiciones de salud ^(26)^.

Si bien existen barreras para trabajar la importancia de la prevención del VIH/sida con la población en cuestión, tales como conductas sexuales de riesgo, escaso conocimiento sobre sexualidad y dificultad o imposibilidad para quedar embarazada, la intervención educativa, a través del juego *Mural de Risco*, demostró ser eficaz para aumentar el conocimiento al inicio y después de la intervención sobre la prevención del VIH/sida en personas de 50 años o más en el contexto escolar.

Sin embargo, este resultado, si bien presenta evidencia positiva, también plantea una reflexión sobre la importancia de invertir en acciones de educación para la salud que fomenten la práctica sexual segura ^(27)^, dado que el acceso al conocimiento sobre la prevención del VIH/sida por sí solo puede no ser suficiente o simplemente no implica necesariamente que va a haber un cambio en las prácticas sexuales sin protección.

En cuanto a la religiosidad, casi la mitad de los participantes eran católicos. Al evaluar la espiritualidad, religiosidad y creencias del público en cuestión, se comprobó, en un estudio similar, que los aspectos espirituales y religiosos están comúnmente presentes en la vida de estas personas. El hecho de que, comúnmente, la religión asocie la sexualidad sólo con el acto sexual en sí y no con la comprensión del mismo en su totalidad, puede ser un factor inhibidor de la práctica sexual en dicho público o, incluso, un factor de riesgo porque puede generar un sentimiento de repulsión ^(28)^.

Se observó, durante el estudio, que además de probar la efectividad del juego, también se destacó la importancia de realizar acciones de educación para la salud sobre temas relacionados a la sexualidad con el público de esta investigación, con el uso de tecnologías educativas, dado que pueden estimular enormemente la cognición mediante el uso de la visión, el oído y el tacto, lo que influye directamente en la comprensión del tema y la motivación para el aprendizaje. También es importante destacar que estas acciones deben ser impulsadas y realizadas por equipos en la educación básica.

En cuanto a los años de estudio, datos similares encontrados en investigaciones sobre el perfil socioepidemiológico y la autonomía de personas longevas ^(29)^, demostraron que el 46,0% de la población tenía entre uno y cuatro años de estudio. Otra investigación, realizada en el norte de Brasil ^(28)^ sobre alfabetización en salud sobre el VIH, encontró que la mayoría de las personas no estaba escolarizada o solo había completado la escuela primaria. Se observa que el bajo nivel de alfabetización favorece que aumente la vulnerabilidad a la infección por VIH y, por ende, es necesario aunar esfuerzos para prestarle mayor atención y darle mayor visibilidad a la atención dirigida a la salud sexual de esta población.

La inactividad sexual que reveló el público objetivo de este estudio coincide con lo identificado en investigaciones que demostraron que factores como la presencia de comorbilidades, cambios corporales, impotencia sexual y una visión social distorsionada de la sexualidad hacen que las personas de 50 años o más no se sientan cómodas o incluso se sientan culpables al expresar sus deseos sexuales ^(30)^. Sin embargo, durante el juego, los estudiantes verbalizaron su deseo e interés sexual.

Dada la exposición física, cultural y sexual al VIH, se comprueba lo importante que es, debido a los datos del perfil sociodemográfico de los participantes del presente estudio, aclarar aspectos relevantes que ayuden a brindar una mejor atención a la salud sexual de las personas mayores. Estos hallazgos pueden contribuir a la atención de enfermería, a través de una mejor planificación y orientación de las directrices, el diálogo, los comportamientos y de la alfabetización para la salud en esta población. Por lo tanto, hay que brindarle un espacio inclusivo y focalizado, con escucha calificada, diálogo libre de juicios, creación de vínculos y prácticas basadas en la evidencia ^(31)^.

La revisión sistemática de ensayos clínicos aleatorizados sobre juegos y enfoques lúdicos en la atención para la salud demostró que la mayoría de los estudios primarios arrojaron como resultado que hubo un aumento significativo del conocimiento mediante el uso de juegos de mesa en comparación con el uso de otros juegos. El metanálisis realizado en esta revisión mostró que el efecto de los juegos de mesa sobre el conocimiento relacionado con la salud fue medio (d* = 0,82, intervalo de confianza del 95%; IC [0,15–1,48]), y que se registró un aumento del conocimiento sobre la salud en la mayoría de las intervenciones que utilizaron juegos de mesa (76,0%, n = 16) ^(32)^.

Al garantizar la efectividad del *Mural de Risco*, se contribuye al cuerpo de conocimiento de la enfermería basada en la evidencia que incluye al paciente en el proceso de atención, que se considera un desafío ^(33 - 34)^. Por consiguiente, la tecnología ha demostrado ser útil para difundir conocimiento sobre la prevención del VIH y favorecer al mismo tiempo el protagonismo en el autocuidado y, por ende, se cree que puede contribuir a reducir las tasas de VIH/SIDA en el público en cuestión.

Por lo tanto, el estudio puede contribuir a la atención de enfermería planificada y centrada en las directrices, el diálogo y los comportamientos de salud para dicha población. Mediante el uso del *Mural de Risco*, una tecnología de bajo costo y fácil acceso, por parte de los enfermeros durante las acciones de educación para la salud, será posible ofrecer un espacio inclusivo dirigido al público de 50 años o más, con escucha calificada, diálogo libre de juicios, creación de vínculos y prácticas basadas en la evidencia.

Por ello, y considerando los resultados que los juegos de mesa tienen sobre el conocimiento en salud, como el conocimiento sobre VIH e ITS, además de las implicaciones que tiene para el avance del conocimiento científico en el área de la salud y la enfermería, se sugiere que los resultados discutidos aquí no se limiten a este estudio.

Se consideran limitaciones del estudio el potencial sesgo de cortesía, por haber aplicado los instrumentos antes y después de un momento lúdico; el hecho de que no se consideraron en el análisis de los datos las diferencias sociales, relacionales y emocionales de las etapas de vida de los participantes; y que la evaluación de la retención de conocimiento después de la intervención propuesta se llevó a cabo sólo de forma inmediata y a medio plazo. Por ello se sugiere que se realicen nuevos estudios analíticos y/o cualitativos para evaluar el fenómeno en cuestión.

Cabe destacar que el público objetivo del estudio se amplió a la franja etaria de 50 años o más, a diferencia del registro en el protocolo ReBEC que consideró la inclusión de personas de 60 años o más, debido a que el perfil epidemiológico actual muestra que hay una alta Tasa de VIH en esta población específica ^(1)^.

## Conclusión

El juego de mesa *Mural de Risco* resultó efectivo para aumentar el conocimiento de las personas de 50 años o más que participaron en las intervenciones educativas. Por lo tanto, puede ser utilizado en la atención de enfermería con personas de 50 años o más, ya sea en el contexto escolar o en los niveles de atención de salud, para que dicha población aprenda a prevenir el contagio del Virus de Inmunodeficiencia Adquirida/sida.

Para identificar vacíos de investigación que, tal vez, no han sido observados, se recomienda realizar nuevas investigaciones que incluyan el tema abordado y el uso de este juego en otros contextos, como personas de 50 años o más que tienen alto nivel educativo.

Por último, cabe resaltar que el juego *Mural de Risco*, tecnología educativa registrada en la biblioteca nacional (número de registro: 878.773 - libro: 1712 - foja: 481), puede ser replicado a bajo costo, incluso de forma artesanal, y hacer que el aprendizaje sobre la prevención del VIH/sida sea más accesible y democrático.
